# Esterified Lignin Nanoparticles for Targeted Chemical Delivery in Plant Protection

**DOI:** 10.1021/acsami.4c16912

**Published:** 2024-12-21

**Authors:** Matilda Andersson, Ievgen V. Pylypchuk, Alexandros Efraim Alexakis, Li-Yang Liu, Mika H. Sipponen

**Affiliations:** † Department of Materials and Environmental Chemistry, Stockholm University, Svante Arrhenius väg 16C, 10691 Stockholm, Sweden

**Keywords:** lignin, esterification, wax interaction, entrapment, model system

## Abstract

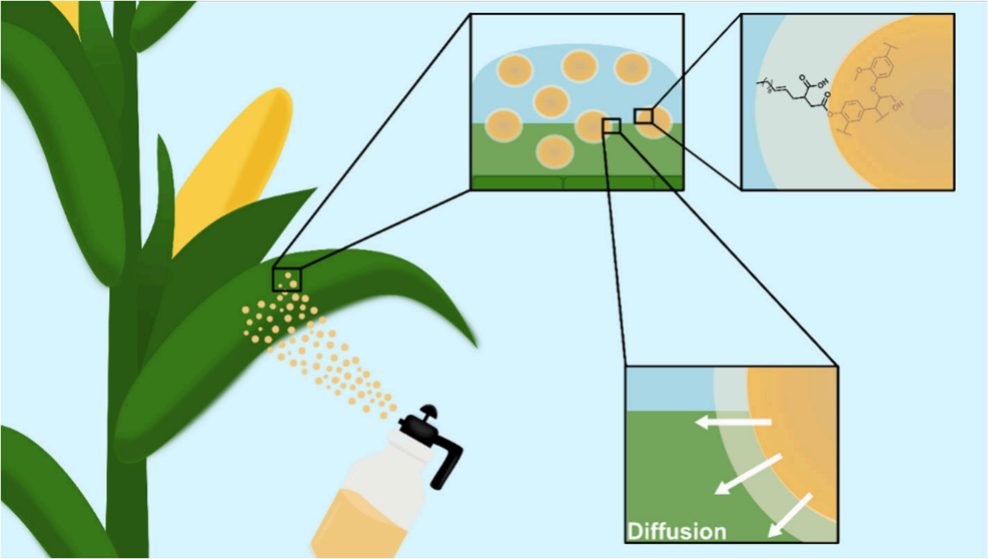

There is a growing demand for biobased functional materials that can ensure targeted pesticide delivery and minimize active ingredient loss in the agricultural sector. In this work, we demonstrated the use of esterified lignin nanoparticles (ELNPs) as carriers and controlled-release agents of hydrophobic compounds. Curcumin was selected as a hydrophobic model compound and was incorporated during ELNP fabrication with entrapment efficiencies exceeding 95%. ELNPs presented a sustained release of curcumin over 60 days in an oil medium, with a tunable release rate dependent on the lignin-to-curcumin mass ratio. The ELNPs showed a strong adhesion interaction with the hydrophobic wax surface. Quartz crystal microbalance with dissipation monitoring (QCM-D) and atomic force microscopy (AFM) analysis suggested that the ELNPs permeated into the wax layer, potentially preventing pesticide loss due to runoff or rainwater leaching. Rapidly decreasing contact angles between a droplet containing an aqueous dispersion of the ELNPs and a fresh leaf surface provided further evidence of a favorable interaction between the two. Overall, our results portray ELNPs as promising biobased nanoparticulate systems for pesticide delivery to hydrophobic plant surfaces.

## 1 Introduction

Global food security faces increasing stress, as the world population continues to rapidly grow. This necessitates more intensive agricultural practices,^1^ leading to an increase in the use of agrochemicals such as pesticides. Particularly chemical pesticides have well-documented negative effects on the nearby environment and human health,^2−4^ necessitating mitigation efforts. To combat this challenge, intergovernmental agencies around the world are working to limit the use of chemical pesticides. One example is the Farm to Fork strategy from the European Commission, which aims to reduce the use of pesticides by 50% by 2030.^5^ Unfortunately, current trends around the world do not align with these efforts. Data from the Food and Agriculture Organization of the United Nations show a steady increase in pesticide use worldwide since 1990,^6^ with countries like Sweden recently selling record-high quantities of pesticides for agricultural purposes since 1991–1995.^7^

Out of the nearly 4 million tonnes of pesticides applied annually, only a very small percentage (1–25%) reaches its intended targets.^8^ This substantial loss is due to factors like spray drift, runoff, rainwater leaching, and degradation by UV radiation.^8,9^ As a countermeasure, encapsulation of active ingredients in nanoparticles with tunable size and surface chemistry has been studied to reduce losses through improved UV shielding and increased foliar adhesion.^8^ Additionally, the increased efficiency of the applied pesticides could contribute to reducing their environmental risk.^9^

Lignin, a major component of wood and a polyphenol, is a common byproduct of biobased industries.^10,11^ Even though lignin is generally burnt for energy purposes,^12^ it has been shown to have great potential as a building block for the development of sustainable materials. For example, kraft lignin, precipitated from black liquor,^13^ has been used as the basis for a wide range of functional materials, including sunscreens,^14^ microfibers for water purification,^15^ and colloidal lignin particle gels,^16^ which have potential applications in 3D printing inks and porous materials.

In view of pesticide delivery, lignins possess lucrative properties like UV absorbance,^17^ antioxidant activity,^18^ and the ability to act as a carbon source for soils.^19^ Furthermore, the hydroxyl groups of lignin allow for straightforward modification through esterification.^20^ These properties have already been recognized in plant protection. For instance, Beckers et al. used methacrylated lignosulfonate to form nanocarriers with hydrophobic cargo via interfacial cross-linking.^21^ Non-cross-linked lignin nanocarriers have been prepared using esterified lignosulfonate and sodium dodecyl sulfate,^22^ and double-shelled lignin nanocapsules formed from hardwood kraft lignin using either cationic or anionic surfactants as a sacrificial template.^23^ Recently, efforts to use fractionated lignin nanoparticles (LNPs) have also been reported. For instance, Liu et al. showcased the possibility of using LNPs from the acetone-soluble lignin fraction for fungicide entrapment. While the results showed efficient protection and reduced acute toxicity against zebrafish,^24^ the important challenge of achieving strong adherence to plant leaves was not investigated.

In the present work, esterified lignin nanoparticles (ELNPs) were explored as a potential biobased carrier system for hydrophobic compounds, such as certain chemical pesticides. Softwood kraft lignin (SKL) was esterified using (2-dodecen-1-yl)succinic anhydride (DDSA), and the resulting esterified lignin (EL) was used to form ELNPs through a solvent-shift methodology. Using curcumin as a hydrophobic model compound, we evaluated how the enhanced lipophilic nature of the lignin nanoparticles influences the entrapment and consequent release of the hydrophobic cargo into a cuticle-like environment. Additionally, the interactions between unloaded ELPNs and wax extracted from *Zea mays* L. were evaluated in both dry and suspension states. Only the ELNPs demonstrated a strong interaction between the nanoparticles and the model surface, allowing for the sustained release of cargo and showcasing the potential of ELNPs as a foliar pesticide carrier for plant protection.

## 2 Results and Discussion

**Figure 1:**
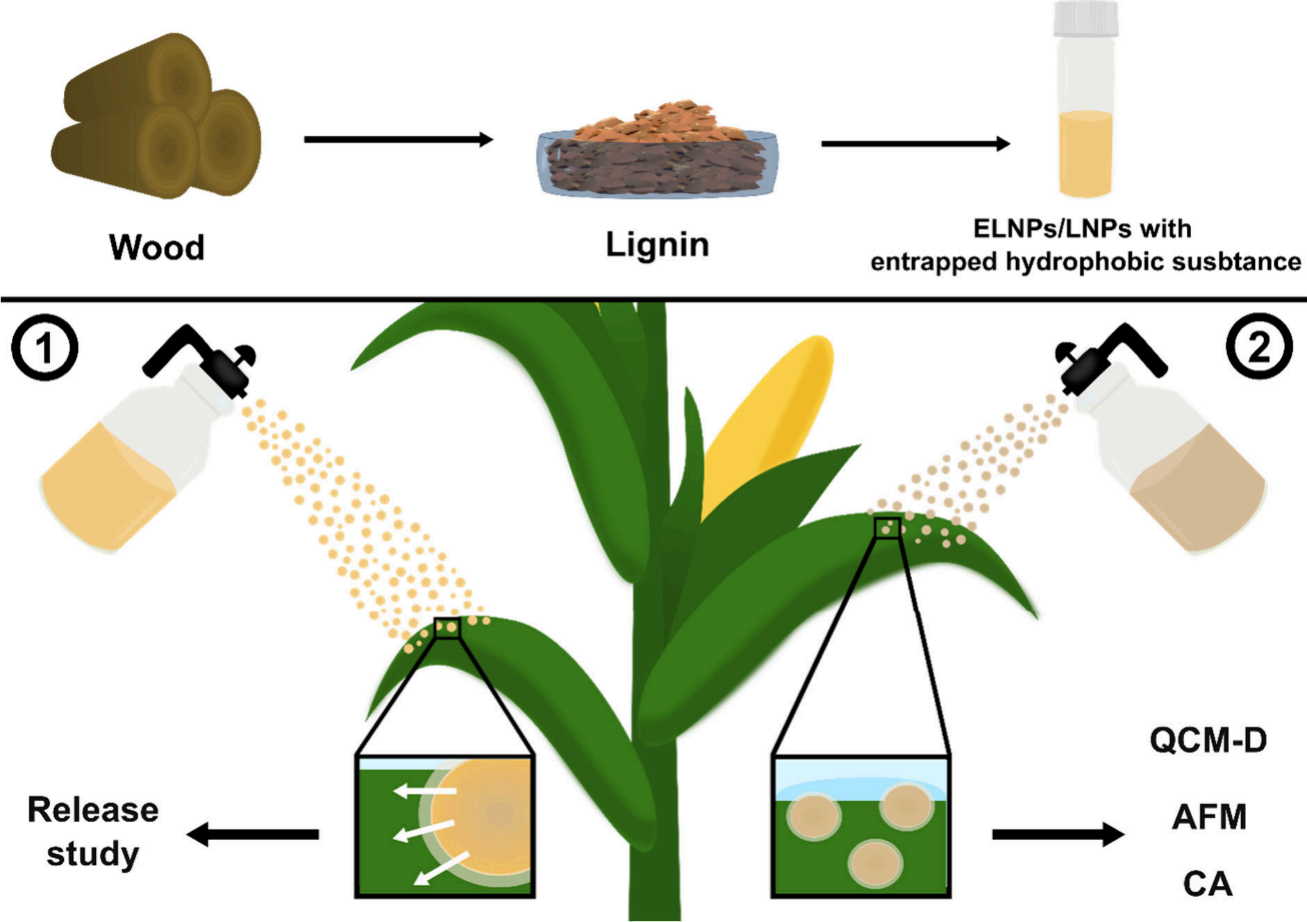
General schematic of the work. Two main objectives were investigated: (1) release of a hydrophobic cargo in a cuticle-like environment and (2) the interaction of the ELNPs on a hydrophobic surface.

The goal of this study was to explore the potential of using ELNPs as a biobased pesticide delivery system. The investigation was focused on two main objectives: (1) the release of hydrophobic cargo in a cuticle-like environment and (2) the study of the nanoparticle interaction with a hydrophobic surface (Figure 1).

**Figure 2:**
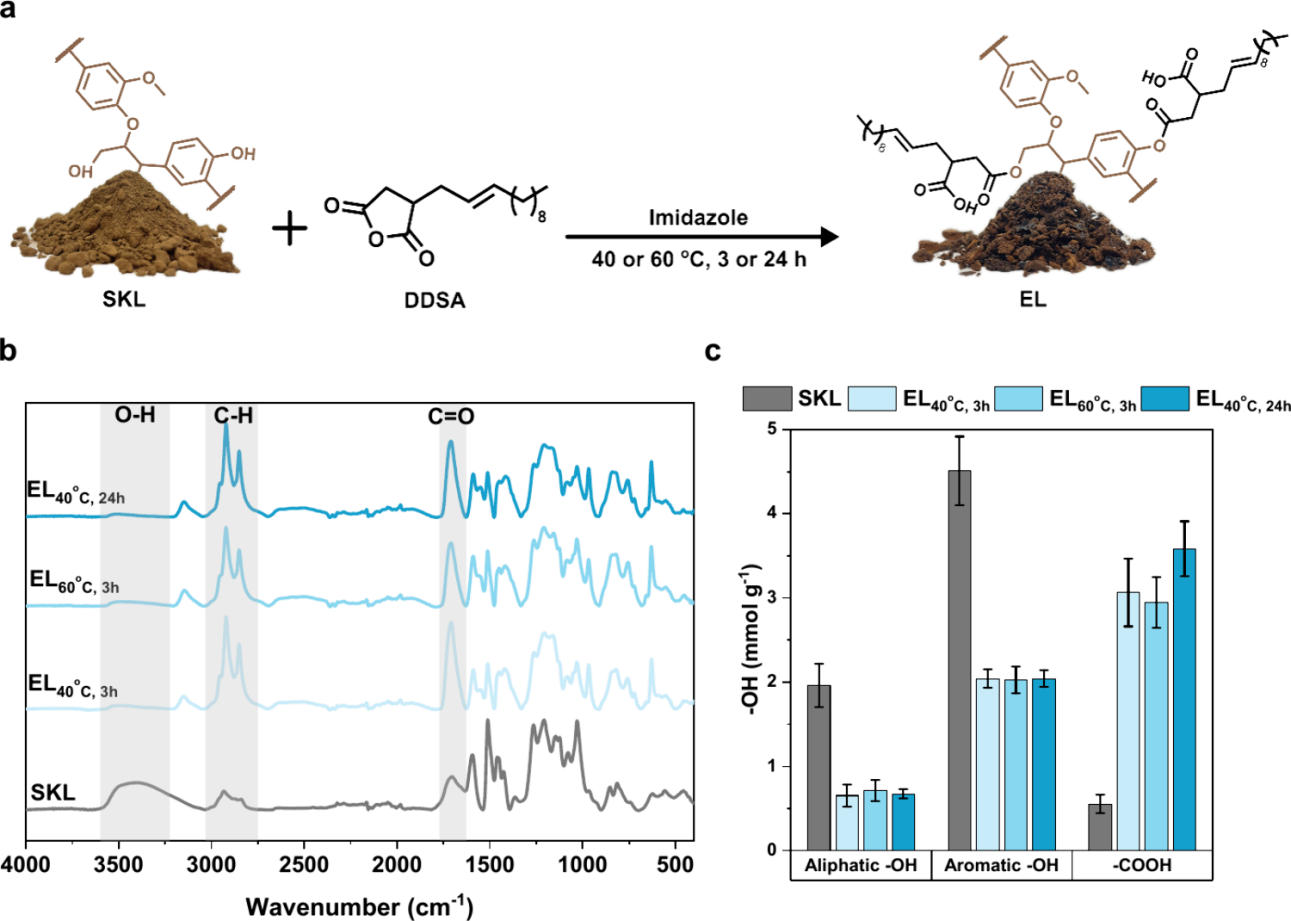
Esterification of SKL with DDSA. (a) Schematic illustration of the experimental procedure of esterification, (b) FTIR spectra, and (c) quantification of the −OH groups on the lignins before and after esterification. Error bars in (c) represent ± one standard deviation from the mean value (*n* = 3).

To esterify the SKL, the fatty acid derivative (2-dodecen-1-yl)succinic anhydride (DDSA) was used to functionalize the surface hydroxyl groups (phenol and aliphatic OH groups) of lligninin acetone/water (3:1 mass ratio) solvent, together with imidazole to facilitate a base-catalyzed esterification (Figure 2a). The Attenuated Total Reflectance Fourier transform Infrared (ATR-FTIR) spectroscopy of SKL before and after esterification at different temperatures and reaction times is presented in Figure 2b. After esterification, the FTIR spectrum of EL shows a decrease in the intensity of the O–H stretching vibration band around 3450 cm^–1^, indicating a successful reaction of the hydroxyl groups in the lignin structure under all tested conditions. Additionally, the increased intensity of the C–H stretching vibrations at 2920–2850 cm^–1^ and the C=O stretching vibration at 1700 cm^–1^ provided evidence of the presence of fatty acid aliphatic chains and ester functional groups in the sample.

Analysis of the hydroxyl and carboxyl groups present on the SKL and EL was performed using quantitative phosphorus-31 nuclear magnetic resonance (^31^P NMR) spectroscopy (Figure 2c). The ^31^P NMR spectroscopy data is presented in Figure S1 and Table S1. Previous reports have found the total hydroxyl group content of SKL to be between 7.76 and 6.67 mmol g^–1^.^25,26^ Herein, the total hydroxyl content, expressed as the sum of phenolic and aliphatic of SKL, was determined to be 6.5 ± 0.7 mmol g^–1^. Comparing the aliphatic and phenolic hydroxyl contents of SKL with the different ELs, a decrease of more than 50% of the hydroxyl groups was observed, indicating the successful esterification reaction. Furthermore, the increased carboxylic acid in esterified lignins proved successful in ring-opening of DDSA. A comparison of the total hydroxyl content between SKL and the three EL samples showed that the degree of esterification was approximately 60%. As the three EL samples showed similar chemical compositions, these fractions were pooled for the preparation of nanoparticles.

**Figure 3:**
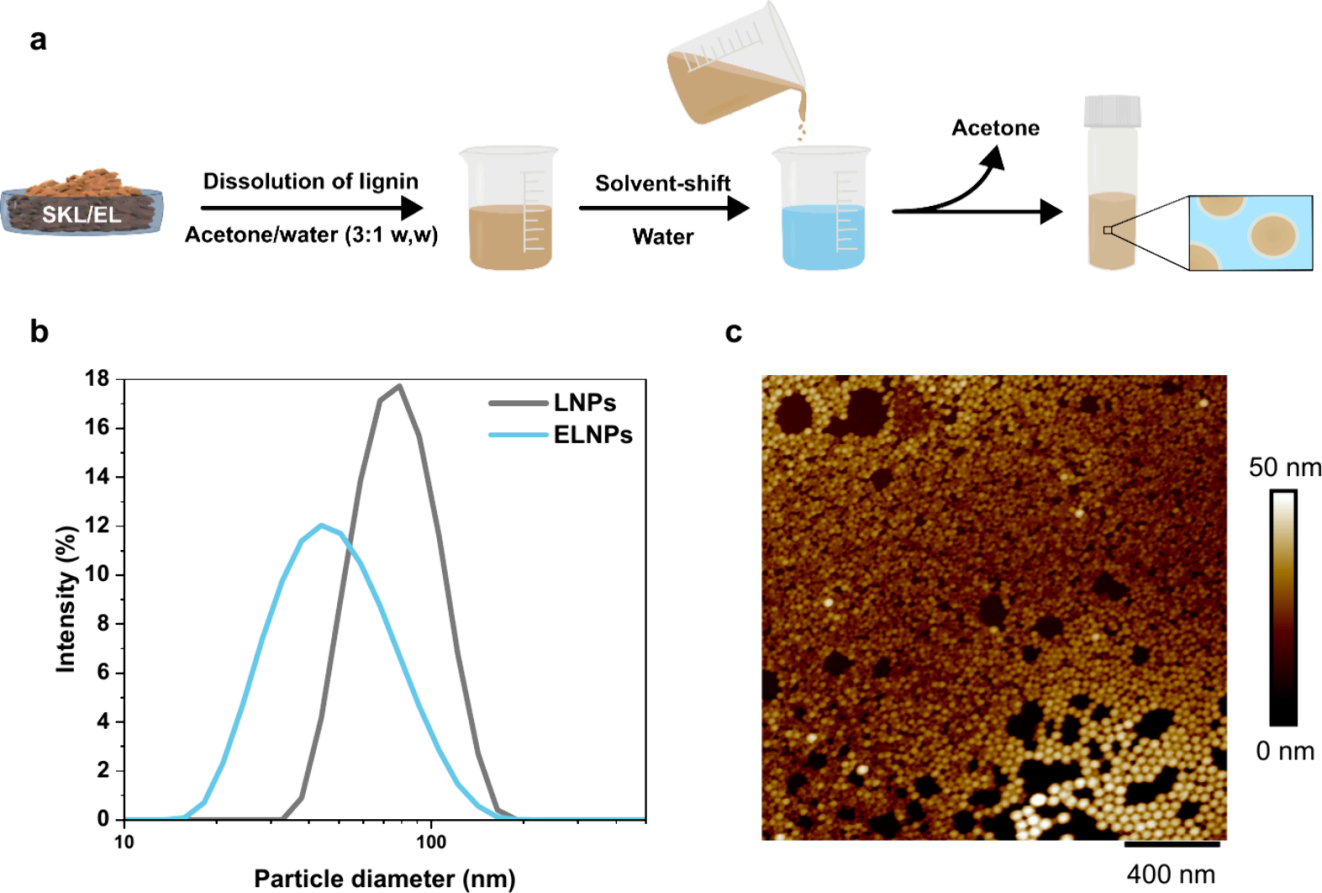
Formation of nanoparticles. (a) Schematic illustration of the synthesis of nanoparticles, (b) size distribution, and (c) a representative AFM-height image of ELNPs.

To assess how the esterification of lignin affects the nanoparticle size and stability, EL and SKL were used for the formation of ELNPs and LNPs using the solvent-shifting methodology (Figure 3a). Determined by dynamic light scattering (DLS), the average hydrodynamic diameter of the ELNPs was 50 ± 6 nm, while LNPs had a diameter of 86 ± 19 nm (Figure 3b). The difference in size between LNPs and ELNPs has previously been explained by Tian et al., who esterified alkali lignin with succinic anhydride.^27^ As a result of the lignin esterification, the lignin–water interaction decreases^27^ and lignin–lignin interaction increases,^28^ and consequently smaller particles are formed from EL than SKL. This observation of particle size differences was supported by AFM imaging, where ELNPs appeared smaller than LNPs (Figure 3c and Figure S2). The ζ-potential values for both ELNPs and LNPs were determined to be approximately −30 mV (Figure S2) despite the large difference in their carboxylic acid contents (Figure 2c), suggesting that even a low concentration of carboxylate groups suffices to render the particle surfaces hydrophilic (Figure S2).

**Figure 4:**
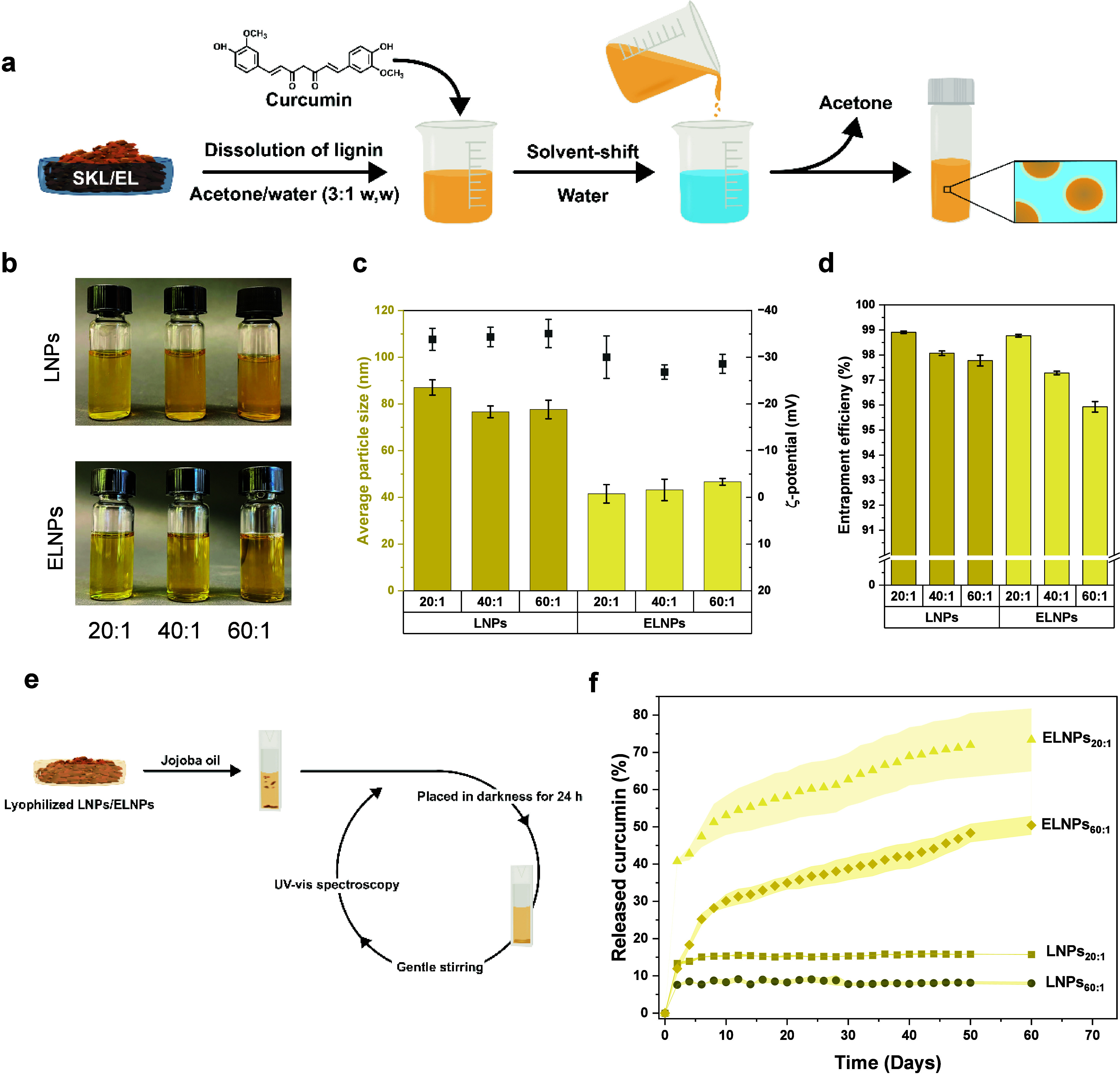
Entrapment of hydrophobic cargo in regular and esterified lignin nanoparticles. (a) Schematic illustration of the synthesis pathway for entrapment of curcumin and (b) digital images of formed particles with different mass ratios of lignin to curcumin. (c) *Z*-average particle size (DLS) and ζ-potential of nanoparticles and (d) entrapment efficiency of curcumin. (e) Schematic of release testing and (f) mean release kinetics of entrapped curcumin from LNPs/ELNPs in jojoba oil based on two replicates. The upper and lower boundary of the shaded area behind the data points in (f) represent the two individual replicates making up the mean. Error bars in (c) and (d) represent ± one standard deviation from the mean values (*n* = 9 and 6 respectively).

Successful entrapment of cargo and subsequent controlled release are two important aspects of an efficient pesticide delivery system. Herein, the hydrophobic compound curcumin was selected to investigate these capabilities. The entrapment was achieved by adding curcumin to dissolved lignin at different concentrations, followed by nanoparticle formation through the solvent-shift approach (Figure 4a). The obtained curcumin-loaded lignin particle dispersions had a yellowish tint as a result of the added curcumin (Figure 4b). The entrapment of curcumin was determined by spectrophotometric measurement, quantifying the unentrapped curcumin present in the supernatant after centrifugation. The results showed that the entrapment efficiency exceeded 95% for both LNPs and ELNPs across all lignin-to-curcumin ratios (Figure 4d). These results compare favorably to previous works that have reported entrapment efficiencies of 91 and 92% using lignin nanoparticles.^29−31^

When the average particle size and ζ-potential (Figure 4c) of the curcumin-containing lignin particle were compared at different lignin-to-curcumin mass ratios, the LNPs also had a larger particle size than ELNPs. ELNPs presented a size of 42 ± 4, 43 ± 5, and 47 ± 2 nm for lignin-to-curcumin mass ratios of 20:1, 40:1, and 60:1 respectively, while the LNPs had a size of 87 ± 3, 77 ± 3, and 78 ± 4 nm for lignin-to-curcumin mass ratios of 20:1, 40:1, and 60:1 respectively. In general, the curcumin entrapment did not significantly alter the ζ-potential or particle size compared to the nanoparticles without curcumin.

When sprayed on plants, it is beneficial if the pesticides absorb into the cuticle, followed by translocation to required areas.^32−34^ Hence, the release of curcumin was tested in a hydrophobic environment chosen to mimic the plant cuticle and without external triggers to promote the release. The epicuticular wax of *Zea mays L.* is reported to be a mixture of mostly esters, along with some alkanes, alcohols, fatty acids, and a small percentage of aldehydes and sterols.^35^ Jojoba oil was selected as a release medium as it is composed mainly of wax esters with some free fatty acids, alcohols, hydrocarbons, sterols, and some triglyceride esters.^36,37^ This makes it more comparable to the epicuticular wax than other natural oils such as palm oil or corn oil, which mainly consist of triglycerides and free fatty acids.^38,39^

Lyophilized LNPs and ELNPs, with lignin-to-curcumin mass ratios of both 20:1 and 60:1, were immersed in jojoba oil for a diffusion study in a dark environment for a total of 60 days (Figure 4e). Due to curcumin’s limited solubility in jojoba oil, a maximum attained release level was determined by sonicating the particles in oil for 1 h. In this solubility-limited environment, the highest curcumin release, 0.32% of the entrapped curcumin, was obtained for ELNPs with a lignin-to-curcumin mass ratio of 20:1. This value was used as the maximum curcumin release level for these systems in jojoba oil (Figure S3).

Interestingly, even though both of the nanoparticle systems showed high entrapment efficiencies, compared to the regular particles the curcumin release from ELNPs was more extensive and sustained over an extended time period of more than 60 days. ELNPs with a mass ratio of 60:1 (lignin to curcumin) released 50% of their oil-soluble cargo, while ELNPs with a mass ratio of 20:1, released 73%. In contrast, LNPs with a mass ratio of 20:1 (lignin to curcumin) plateaued after 6 days at a level of 15% (Figure 4f). LNPs with a mass ratio of 60:1 showed an even lower release of 8.5% and plateaued after 4 days. The sustained release of curcumin from ELNPs contributes to prolonged and steady protection, highlighting a substantial difference in the release mechanisms between regular LNPs and ELNPs despite their similar entrapment efficiencies. We speculate that the high amphiphilicity of ELNPs originating from the inserted alkyl chains and associated carboxylic acid groups is a likely reason for the preferred diffusion of curcumin from the particles. A sustained release allows for efficient uptake by the plant and reduced amount of active ingredient needed when compared to bulk formulations.^40^

The difference in release kinetics between the two nanoparticle systems is probably attributed to the aforementioned differences in the chemical structures. Previous reports have shown that esterified lignin nanoparticles have the alkyl chains of the fatty acids residing close to the particle surface.^41^ This would contribute to a more lipophilic particle surface and better interaction with the jojoba oil. Furthermore, the release profiles of both the LNPs and ELNPs demonstrated that an increased lignin-to-curcumin ratio contributes to a slower release of the cargo. Therefore, these results indicate that the system can be optimized, depending on the specific plant and pesticide.

Release kinetics and the underlying mechanism were investigated using several kinetic models (Figure S4 and Table S2). The correlation coefficient (*R*^2^) values suggested that the Higuchi model provided the best fit for ELNPs with a mass ratio of 20:1, while the Baker-Lonsdale model appeared to be the best fit for ELNPs with a mass ratio of 60:1. These results suggest that the release mechanism for both systems is based on Fickian diffusion.^42^ Moreover, the Korsmeyer-Peppas model presented *n* values below 0.43 for both of the evaluated lignin-to-curcumin ratios, further supporting the release driven by Fickian diffusion.^43^ Thus, the release of cargo occurs due to the difference in chemical potential between the nanoparticles and oil medium and not as a result of swelling or erosion of the nanoparticles.

A comparison of the obtained release after 30 days with samples placed under stirring during the same period presented a clear increase in the extent of cargo release. The released mass from the ELNPs increased by 46% and for the LNPs the increase was 52% due to the stirring that facilitated mass transport. Moreover, an increase in oil volume from 2 to 5 mL resulted in an increased release of 33% for the ELNPs (Figure S3). These results further strengthen the modeling results, indicating that Fickian diffusion is driving the release.

**Figure 5:**
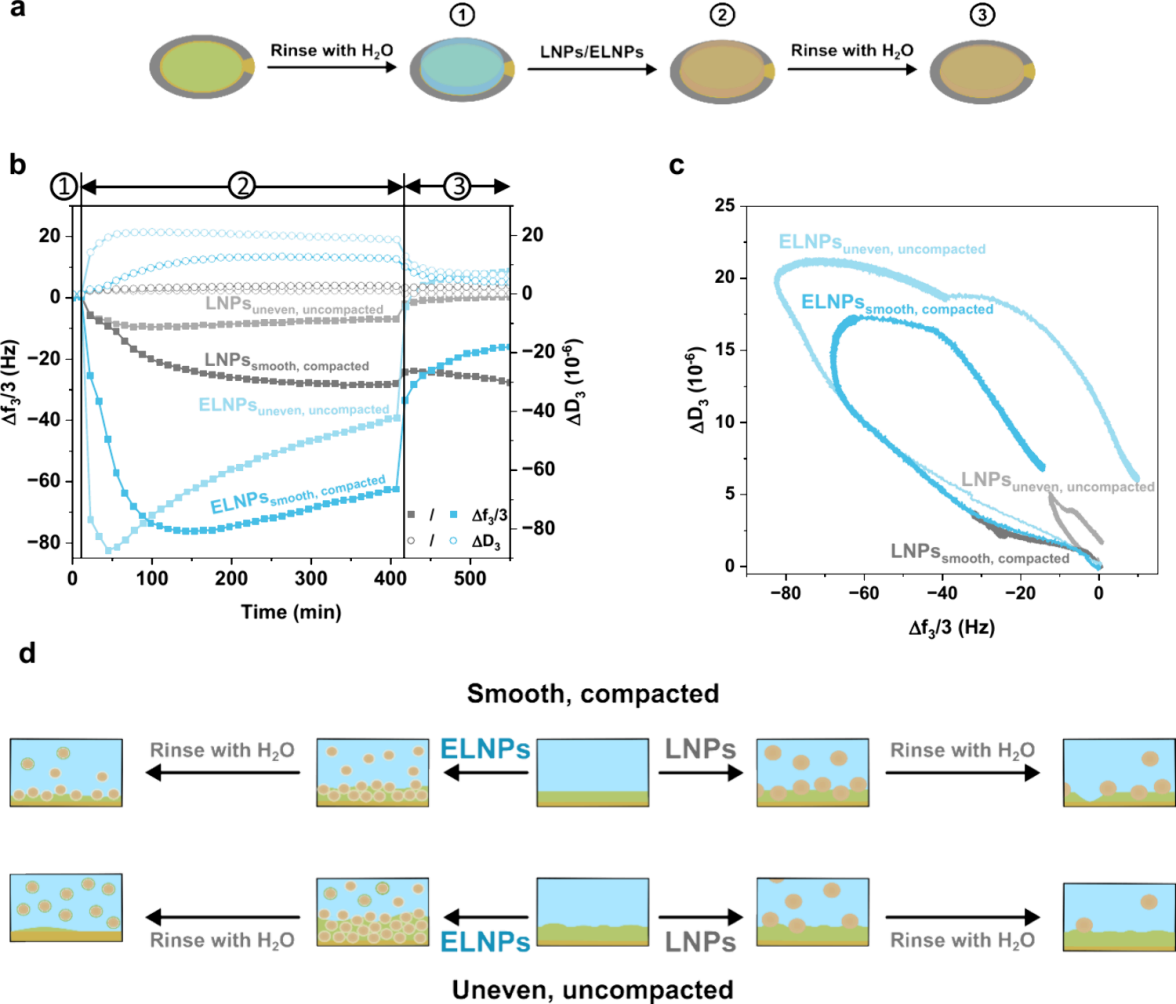
Nanoparticle interaction with the wax surface. (a) Schematic illustration of the general procedure for QCM-D analysis with corresponding (b) frequency (Δ*f*_3_/3) and dissipation (Δ*D*_3_) results for the third overtone. (c) Dissipation vs frequency plot and (d) graphical interpretation of the obtained results. Samples noted with “smooth, compacted” refers to the spin coated and melted wax surface. Samples noted as “uneven, uncompacted” refers to a spin coated wax surface.

One of the most important aspects of a foliar pesticide delivery system is its ability to interact favorably with the cuticular wax and, ideally, permeate through the hydrophobic outer layers.^44^ To evaluate this, QCM-D was employed using gold sensors coated with extracted wax from the leaves of *Zea mays L*. A baseline was established in deionized water, followed by the addition of an aqueous dispersion with the different nanoparticles, and finally, the sensor was rinsed with deionized water (Figure 5a).

When nanoparticles are adsorbed onto the surface of the sensor, a negative shift in the frequency is observed, corresponding to an increased mass (Figure 5b). It is well-known that the larger the shift, the more mass added to the sensor. The dissipation shows a positive shift, indicating the formation of a viscoelastic layer, while no or just a slight shift indicates a rigid layer. By visualizing the relationships between shifts in frequency and dissipation for the different surfaces and particles, it is possible to get a clear overview of the interaction (Figure 5c). It is evident that the ELNPs have a higher affinity toward the wax surface compared to the LNPs. Additionally, based on the differences in changes in dissipation the particles formed different types of layers on the wax, with ELNPs depositing less dense layers compared to the ones from the LNPs.

Two different approaches were employed for preparing the sensors. To start, all sensors were spin-coated with extracted wax. Subsequently, half of the sensors were exposed to the melting temperature of the wax for 5 min to ensure a uniform coating, while the other half was held at room temperature. The melted sensors appeared to have a smoother and more compact surface compared to the thermally untreated sensors (Figure S7). Thus, the surfaces were noted as smooth, compacted, and uneven, uncompacted for the melted and untreated surfaces, respectively. For the interaction of ELNPs on the uneven, uncompacted wax surface, a strong shift in both frequency and dissipation occurred upon nanoparticle addition (Figure 5c). These results suggest a high attraction between the nanoparticles and the wax surface and swelling of the layer as particles are added. Moreover, as ELNPs interacted with the wax, a maximum frequency change was reached, but thereafter, the frequency started to increase, suggesting mass removal from the surface. As there was still a flow of ELNPs being added onto the sensor, the mass removed was likely not only nanoparticles but also the wax coating. This observation, together with the results from nanoparticle interaction with a clean surface (Figure S6), suggest that the nanoparticles are not only residing on top of the coating but were interacting with the wax, causing the swelling, and the slow removal of mass due to disruption of the wax coating (Figure 5d). Subsequent rinsing with water showed a slight positive frequency shift, indicating that part of the coating was likely removed. Conversely, a small frequency and dissipation shift was observed for LNPs interacting with the uneven, uncompacted wax surface (Figure 5c), indicating weak adhesion of the LNPs to the wax. Upon rinsing with water, the frequency and dissipation values returned close to the baseline, indicating weak interfacial interactions resulting in the removal of most LNPs (Figure 5d).

Regarding the ELNPs’ interaction with the smooth, compacted wax coating, it is again notable that the layer swelled as the ELNPs were added, indicating a high attraction between the particles and the surface (Figure 5c). The attraction and swelling were less prominent than for the uneven, uncompacted surface, likely due to the slightly denser packing of the melted coating (Figure 5d). Nevertheless, the interaction with the wax remained stronger for ELNPs compared to that of LNPs. Furthermore, when comparing the uneven, uncompacted wax surface with the smooth, compacted surface, it seems that upon rinsing with water, nanoparticles still remained attached to the smooth, compacted surface. This could suggest that this surface might be more resistant to disruption compared with the uneven, uncompacted wax surface.

Only a small frequency and dissipation shift was observed for the LNPs on the smooth, compacted wax coating (Figure 5c), again showing a weak adhesion interaction between the surface and LNPs. Nevertheless, during rinsing, the frequency initially increased and then decreased again (Figure 5b). A plausible explanation for this is that the small amount of amphiphilic LNPs that interact with the surface disrupts the coating, generating an uncoated area. As the water rinses away the LNPs, the disrupted area allows water penetration, causing the increased frequency (Figure 5d). Looking at the QCM-D curves of the LNPs and ELNPs with a clean, uncoated surface (Figure S6), the strong interaction and swelling behavior seen with the coated sensor were not observed. Thus, comparing the QCM-D results of coated sensors with clean uncoated sensors, it is evident that the observed mass uptake occurred due to the uptake of the nanoparticles into the wax.

**Figure 6:**
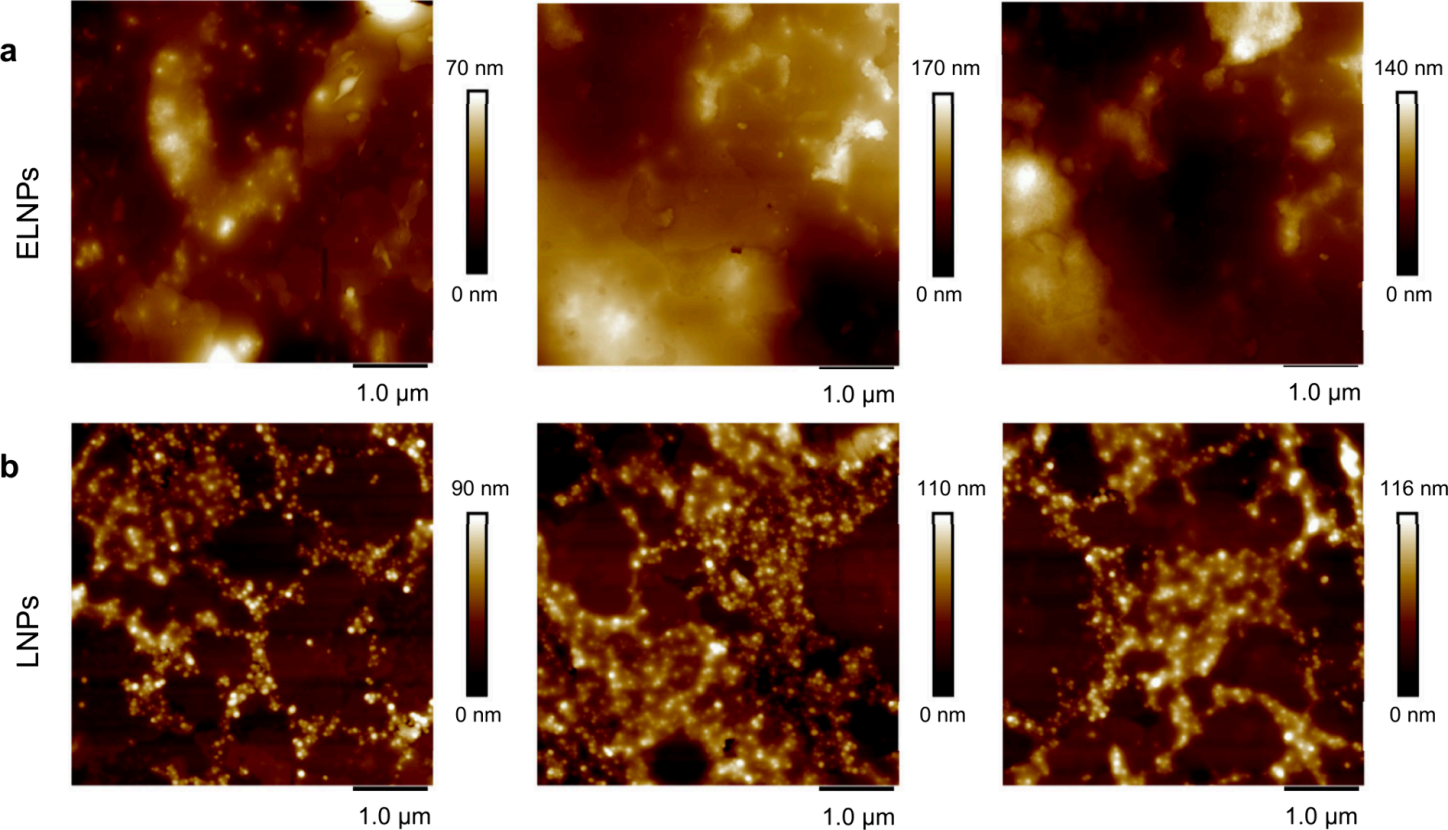
Visualization of nanoparticle adsorption and penetration into the wax layer. AFM-height images acquired in dry state from (a) ELNPs and (b) LNPs on a wax coated surface at different parts of the sample.

AFM was employed to further investigate the nanoparticle uptake and migration into the wax layer, this time in a dry state. The AFM-height images showed that after water evaporation, the ELNPs appeared to fully permeate the wax film (Figure 6a), while only a fraction of the LNPs did (Figure 6b). Though chemically distinct, other materials like gold nanoparticles,^45^ iron–carbon nanoparticles,^46^ and carbon dots^47^ have all exhibited the ability to permeate the leaf surface. Considering this, together with the AFM-height images and QCM-D data, it is plausible that swelling is indeed occurring because of the ELNPs permeating across the surface. The slight swelling observed for LNPs further support this behavior, but with fewer particles penetrating the surface, the dissipation shift in QCM-D analysis was smaller compared to ELNPs.

**Figure 7:**
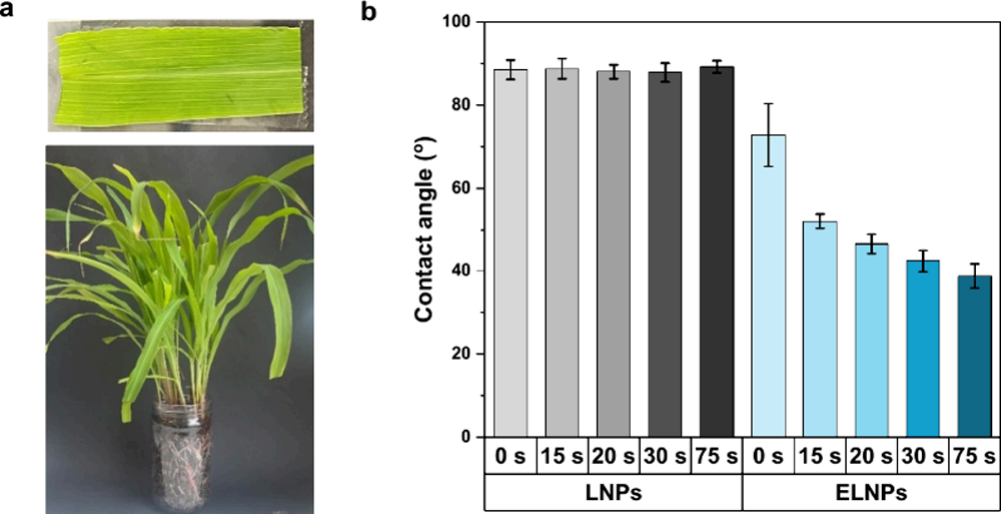
Nanoparticle interaction with leaf. (a) Preparation of surface used for CA measurement using 23-day-old corn leaves. (b) CA measurements for LNPs and ELNPs on the same part of the leaf. Error bars in b represent ± one standard deviation from the mean value (*n* = 5).

The outermost part of the leaf, the cuticle, has a section called epicuticular wax. As formerly stated, this section is composed of a range of long-chained waxy copounds.^35^ Moreover, as previously mentioned, esterified lignin nanoparticles have alkyl chains near their surface.^41^ Thus, the presence of such collapsed alkyl chains probably increased the lipophilic nature of the ELNPs and favorable adherence interactions with the wax. To evaluate this hypothesis, the contact angle (CA) of aqueous lignin nanoparticle dispersions was measured on real corn leaves fixed to microscope slides (Figure 7a). As shown in Figure 7b, there was a notable difference between the two types of lignin nanoparticles. LNPs presented a CA of 88 ± 2.3°, which remained steady during 75 s. In contrast, the ELNPs showed marked wettability, with the CA decreasing from 73 ± 7.6 to 39 ± 2.9° within 75 s. This trend was consistent across three different areas of the leaf surface (Figure S9). Consequently, esterification improved the interaction between the leaf surface and the nanoparticles, potentially enabling permeation upon water evaporation and reducing electrostatic repulsion. These results support the hypothesis that the addition of alkyl chains on the surface of the particles facilitates adsorption onto and qubsequent absorption of ELNPs into the waxy compounds of the leaf.

## 3 Conclusions

This work presented esterified lignin nanoparticles as viable nanocarriers for pesticide delivery to plant leaves. The esterification prior to the entrapment of curcumin increased the extent of release and leaf-particle adherence compared to those of regular LNPs. Curcumin-loaded ELNPs showed a tunable release rate depending on the lignin-to-curcumin mass ratio and exhibited sustained release during 60 days through Fickian diffusion. This sustained release could provide prolonged plant protection, maximizing the cargo efficiency. Investigation of nanoparticle–wax surface interactions using QCM-D and AFM indicated that esterification facilitated permeation of ELNPs through the hydrophobic wax layer. Furthermore, contact angle measurements demonstrated rapid wettability of the ELNPs on an authentic leaf surface in contrast to non-wettability with regular LNPs. Overall, these results demonstrate the potential of ELNPs for reducing pesticide loss due to factors like rain leaching or runoff, offering a promising biobased nanoparticulate system for plant protection.

## 4 Experimental Section

### 4.1 Materials

Softwood kraft lignin (SKL) was purchased from UPM (BioPiva100, Finland), and dried at 50 °C for 12 h before use. Imidazole was purchased from Apollo Scientific. (2-Dodecen-1-yl)succinic anhydride (DDSA), curcumin, and jojoba oil were purchased from Sigma-Aldrich. Acetone and cyclohexane were purchased from VWR. All chemicals were used as received. The corn leaves were collected from Slottstradgrden Ulriksdal in the Stockholm region, Sweden. Deionized (DI) water was used during the experiments. For dialysis, Spectra/Por membranes with MWCO of 12–14 kDa were used and performed against DI water.

### 4.2 Esterification of Kraft Lignin

The esterification procedure was adapted from previous work by Beaumont et al.^48^ and Li et al.,^49^ with slight modifications. DDSA was selected for esterification due to its greener profile compared to fatty acid chlorides, which involve toxic chlorinating agents and harsher solvents. In short, 4.0 g of SKL (containing 6.5 mmol g^–1^ of total phenolic and aliphatic hydroxyl groups based on average from three replicates of quantitative ^31^P NMR) was dissolved in 30 mL of acetone (7.5 mL g^–1^ of lignin) and 10 mL of DI water (2.5 mL g^–1^ of lignin). Thereafter, 1.64 g of imidazole (0.93 molar ratio to the amount of phenolic and aliphatic hydroxyl groups in SKL) and 4.26 g of DDSA (0.62 molar ratio to the amount of phenolic and aliphatic hydroxyl groups in SKL) were added to the mixture and left under stirring at 40 or 60 °C for 3 or 24 h. The product was thereafter dialyzed for 24 h and then dried at 60 °C to obtain a solid powder of esterified product (EL). After analysis with ATR-FTIR, ^31^P NMR, and SEM-EDS (Figure S1) little to no difference could be detected between the batches of EL at different times and temperature. Therefore, the esterified products were considered to have the same inherent properties and were treated as a single batch of esterified product for further experiments.

### 4.3 Preparation of Lignin Nanoparticles

The preparation of all lignin nanoparticles was done using a solvent-shift methodology. Briefly, SKL or EL were dissolved in acetone/water mixture with a 3:1 mass ratio. Undissolved impurities were removed by filtration, and the filtrate was then added to DI water under vigorous stirring to produce the lignin nanoparticles (LNPs) and esterified lignin nanoparticles (ELNPs). Finally, the particles were concentrated by using rotary evaporation.

### 4.4 Entrapment and Release Testing

#### 4.4.1 Entrapment of Hydrophobic Cargo

Curcumin was selected as the hydrophobic cargo for evaluating the encapsulation of the LNPs and ELNPs. For this, the solvent-shift methodology was once again employed. To start, the SKL and EL (2, 4, and 6 g L^–1^) were dissolved in acetone/water (mass ratio 3:1) for 3 h at room temperature, followed by filtration to remove any undissolved impurities. Curcumin was added to the dissolved lignin to achieve a concentration of 0.1 g L^–1^ and left stirring for 5 min. The lignin and curcumin solutions were then quickly added to DI water under vigorous stirring. The formed lignin nanoparticles were dialyzed for 24 h, changing DI water intermittently.

Evaluation of entrapment efficiency was based on previous work by Beckers et al.,^21^ herein with slight modifications. In brief, 2 mL of fresh lignin nanoparticles with entrapped curcumin was centrifuged at 13 400 rpm for 90 min. Thereafter, acetone was added to 1 mL of supernatant (mass ratio 3:1 acetone/supernatant) and analyzed using Genesys 150 UV–vis spectroscopy (Thermoscientific, USA) λ = 425 nm. The mass of not entrapped curcumin was determined using a calibration curve of curcumin in acetone/DI water (3:1 mass ratio) (Figure S3). Subsequently, EE was calculated according to the equation
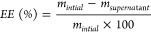
1where *m*_initial_ is the initial mass of curcumin added to the lignin solution and *m*_supernatant_ is the mass of curcumin found in the supernatant after the formation of particles.

#### 4.4.2 Release Kinetics of Hydrophobic Cargo in Jojoba Oil

The release kinetics in a wax-like environment were evaluated by allowing 2 mg of lyophilized LNPs and ELNPs, with entrapped curcumin and lignin-to-curcumin mass ratios of 20:1 or 60:1, to sit in 2 mL of jojoba oil in a dark environment at room temperature. The release was monitored by UV–vis spectroscopy measurements (Genesys 150, Thermoscientific, USA) taken at set time intervals at λ = 416 nm. Before each measurement, the jojoba oil was gently mixed to homogenize the mixture. The percent of release curcumin was calculated using the formula
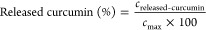
2where *c*_released-curcumin_ is calculated using a calibration curve for curcumin in jojoba oil (Figure S3) and *c*_max_ is given by the maximum obtained release after 1 h of sonication of 2 mg of lyophilized particles in 2 mL of jojoba oil.

Control experiments of the release were also performed using minimal stirring. The same mass of particles and volume of jojoba oil was placed in cuvettes, covered from sunlight, and allowed to stir for 30 days. Thereafter, the samples were centrifuged at 13 400 rpm for 30 min, and the supernatant was subsequently analyzed using UV–vis spectroscopy (λ = 416 nm). The corresponding released mass was calculated using the calibration curve of curcumin in jojoba oil.

Additionally, the investigation of volume dependence was tested by allowing 2 mg of the same ELNPs to release in 5 mL of jojoba oil. After 30 days, UV–vis spectroscopy (λ = 416 nm) was used to calculate the mass of curcumin released using a calibration curve of curcumin in jojoba oil.

#### 4.4.3 Investigation of Release Kinetics

The data were fit to different release kinetics models, such as first-order, Higuchi, Hixson-Crowell, Korsmeyer-Peppas, and Baker-Lonsdale models. This was done by plotting the data according to the different equations to obtain a linear relationship, as previously described.^42,50,51^ Each model was then evaluated based on its correlation coefficient (*R*^2^) to determine which model most accurately described the release. Additionally, for Korsmeyer-Peppas model, the *n* exponent gives insights into the release mechanism. Generally, for spherical systems, if *n* ≤ 0.43, then the release mechanism is Fickian diffusion. If 0.43 < *n* < 0.85, then it indicates anomalous transport. If *n* = 0.85, then it is Case I transport; finally, for *n* > 0.85, the release mechanism is super Case II transport.^43^

### 4.5 Nanoparticle Interaction with Wax

#### 4.5.1 Quartz Crystal Microbalance with Dissipation Monitoring (QCM-D)

The interactions between LNPs/ELNPs and a wax-containing film were studied by using QCM-D E4 (Bioscientific, Sweden). QCM-D allows simultaneous monitoring of changes in resonance frequency and dissipation as matter adsorbs on the model surface deposited on the quartz crystal. By monitoring multiple overtones (*n* = 1, 3, 5, 7, 9, 11, and 13) of the resonance frequency, one can make observations into particular vertical regions of the deposited film. The shift in frequency relates to the change in mass, while changes in dissipation indicate the formation of a rigid or viscoelastic layer. For a rigid layer formation, a small to no change in dissipation would be observed, and for a viscoelastic layer there would be a significant change in dissipation. Further indications of the layer formation can be noted from the behavior of the different overtones monitored. A rigid layer formation commonly shows no strong differences between the overtones for both frequency and dissipation. While a viscoelastic layer would present spreading among the overtones.^52^

Herein, gold sensors (Bioscientific, QSX-301) were cleaned by following the cleaning protocol from the manufacturer and then used for all measurements. The coating of the sensors was adapted from Zhao et al.,^53^ using wax extracted from dried *Zea mays L.* leaves dissolved in cyclohexane (0.5 wt %) using a spin-coater (SPIN-1200T, Midas System) at 3000 rpm for 30 s. Inspired by the work of Fagerström et al.,^54^ the sensors were then placed in the oven (70 °C, 5 min) to allow the wax to melt and form a compacted film. Alternatively, the sensors were left at room temperature overnight to allow for the evaporation of residual solvents. Extraction yield and chlorophyll content, characterization of waxy compounds, AFM images of the coated surface, and an estimation of the coating thickness are presented in the Figures S5 and S7. Chlorophyll content was calculated by using formula S1. Each experiment started with a 10 min baseline using DI water, thereafter the different nanoparticles (0.1 g L^–1^) was added, and after approximately 405 min the system was again rinsed with DI water until a total time of 540 min was reached. The third overtone was monitored for all experiments with a flow rate of 0.1 mL min^–1^ and temperature of 22 °C. Each sample was run in duplicate. Additionally, the LNPs and ELNPs interaction with uncoated gold sensors were also evaluated in duplicates.

#### 4.5.2 Atomic Force Microscopy (AFM)

Silicon wafers were cleaned and subsequently coated with the same wax solution by spin coating as that made for the QCM-D sensors. The silicon wafers were not cured. 5 μL of LNPs and ELNPs were drop-casted onto the wax-coated wafer and left overnight to evaporate. The samples were imaged using a Multimode-8 AFM instrument (Bruker, USA) in peak-force tapping mode with the ScanAsyst automatic optimization algorithm from the manufacturer and a ScanAsyst-Air probe. Images of coated silicon wafers without nanoparticles are presented in Figure S8. The images were processed using NanoScope Analysis 2.0 software (Bruker, USA).

### 4.6 Contact Angle (CA) Measurements

Leaves from a 23-day-old corn plant were cut, fixed to a microscope slide, and used as the test surface for CA measurements. 5 μL of LNPs or ELNPs (0.5 g L^–1^) were added to the surface using Drop Shape Analyzer DSA25E (KRÜSS instruments, Germany). Water was used as a control (Figure S9). Measurements were recorded and analyzed using ADVANCE software (KRÜSS instruments, Germany).

Water contact angle measurements was performed on coatings made from LNP and ELNP dispersions (1 g L^–1^). The dispersions were drop-cast in several layers, allowing for water evaporation between each layer. 1 μL of water was added to the surface using the Drop Shape Analyzer DSA25E. Measurements were again recorded and analyzed using ADVANCE software.

### 4.7 Characterization

Analysis of functional groups in SKL and the esterified products was conducted by using Attenuated Total Reflectance Fourier Transform Infrared (ATR-FTIR) Spectroscopy. For this, a Varian 610-IR Spectrometer with a diamond ATR Optics were used. Measurements were taken from 400 to 4000 cm^–1^ with a total of 32 scans.

Phosphorus-31 nuclear magnetic resonance (^31^P NMR) spectra were recorded and analyzed using a previously described method.^15,55−57^ Briefly, DMF, pyridine, the internal standard *endo*-*N*-hydroxy-5-norbornene-2,3-dicarboximide, the relaxation agent Cr(aca)_3_, the phosphitylation agent 2-chloro-4,4,5,5-tetramethyl-1,2,3-dioxaphospholane (TMDP), and deuterated chloroform was added to dissolve and derivatize the dry lignin. The mixture was then left to stir for 10 min and transferred to an NMR tube. The spectra were recorded within 1 h from the start of sample preparation using a 500 MHz spectrometer (Bruker BioSpin GmbH) 202.47 MHz for ^31^P nucleus. Three replicates of each lignin were analyzed.

The composition of SKL and the three EL batches was investigated using HITACHI-TM3000 Scanning Electron Microscope (SEM) through energy dispersive X-ray spectroscopic (EDS) mapping, obtained on the SEM with Bruker Quantax 70 EDX Spectrometer.

Dynamic light scattering (DLS) was used for analysis of particle size and zeta potential of the synthesized lignin nanoparticles. The measurements were performed using a Zetasizer Nano ZS (Malvern, UK). For zeta potential measurement, a dip cell probe was used. Three measurements for each sample were performed.

AFM was used for imaging the LNPs and ELNPs, as well as silicon wafers and QCM-D sensors coated with wax. For the LNPs and ELNPs, 20 μL of each sample was drop casted onto a cleaned silicon wafer and left to evaporate before analysis. Same settings and tips were used as for the AFM conducted using wax coated wafers with nanoparticles added.

## Supporting Information Available

Experimental procedures for extraction and characterization of wax, ^31^P NMR data, SEM-EDS of SKL and EL, ζ-potential of LNPs and ELNPs, AFM-height image of LNPs, wax coated sensor and silicon wafers, max release in jojoba oil, release testing in larger volumes or with stirring, model testing of release, QCM-D results on clean gold sensor, contact angle with water, and ELNPs contact angle of different areas of the leaf (PDF)

## References

[ref1] The Future of Food and Agriculture: Trends and Challenges; Food and Agriculture Organization of the United Nations: Rome, 2017.

[ref2] Hakeem K. R.; Akhtar M. S.; Abdullah S. N. A. Plant, Soil and Microbes; Springer International Publishing: Cham, 2016.

[ref3] Pathak V. M.; Verma V. K.; Rawat B. S.; Kaur B.; Babu N.; Sharma A.; Dewali S.; Yadav M.; Kumari R.; Singh S.; Mohapatra A.; Pandey V.; Rana N.; Cunill J. M. Current Status of Pesticide Effects on Environment, Human Health and It’s Eco-Friendly Management as Bioremediation: A Comprehensive Review. Front. Microbiol. 2022, 13, 962619 10.3389/fmicb.2022.962619. 36060785 PMC9428564

[ref4] Kaur R.; Choudhary D.; Bali S.; Bandral S. S.; Singh V.; Ahmad M. A.; Rani N.; Singh T. G.; Chandrasekaran B. Pesticides: An Alarming Detrimental to Health and Environment. Science of The Total Environment 2024, 915, 170113 10.1016/j.scitotenv.2024.170113. 38232846

[ref5] European Commission. Farm to Fork Strategy, 2020. https://food.ec.europa.eu/horizontal-topics/farm-fork-strategy_en.

[ref6] FAO. Pesticides Use and Trade, 1990–2021. FAOSTAT Analytical Briefs Series No. 70, 2023. DOI: 10.4060/cc6958en.

[ref7] Edvardsson E. Försålda kvantiteter av bekämpningsmedel 2022; Kemikalieinspektionen: Sundbyberg, 2023. https://www.kemi.se/webdav/files/Kemikaliestatistik/Bek%C3%A4mpningsmedel/forsalda_bkm_2022.pdf (accessed 2024–June–10).

[ref8] Wang D.; Saleh N. B.; Byro A.; Zepp R.; Sahle-Demessie E.; Luxton T. P.; Ho K. T.; Burgess R. M.; Flury M.; White J. C.; Su C. Nano-Enabled Pesticides for Sustainable Agriculture and Global Food Security. Nat. Nanotechnol. 2022, 17 (4), 347–360. 10.1038/s41565-022-01082-8. 35332293 PMC9774002

[ref9] He J.; Li J.; Gao Y.; He X.; Hao G. Nano-Based Smart Formulations: A Potential Solution to the Hazardous Effects of Pesticide on the Environment. Journal of Hazardous Materials 2023, 456, 131599 10.1016/j.jhazmat.2023.131599. 37210783

[ref10] Rajesh Banu J.; Kavitha S.; Yukesh Kannah R.; Poornima Devi T.; Gunasekaran M.; Kim S.-H.; Kumar G. A Review on Biopolymer Production via Lignin Valorization. Bioresour. Technol. 2019, 290, 121790 10.1016/j.biortech.2019.121790. 31350071

[ref11] Kai D.; Tan M. J.; Chee P. L.; Chua Y. K.; Yap Y. L.; Loh X. J. Towards Lignin-Based Functional Materials in a Sustainable World. Green Chem. 2016, 18 (5), 1175–1200. 10.1039/C5GC02616D.

[ref12] Xu J.; Li C.; Dai L.; Xu C.; Zhong Y.; Yu F.; Si C. Biomass Fractionation and Lignin Fractionation towards Lignin Valorization. ChemSusChem 2020, 13 (17), 4284–4295. 10.1002/cssc.202001491. 32672385

[ref13] Tomani P. The LignoBoost Process. Cellul. Chem. Technol. 2010, 44, 53–58.

[ref14] Qian Y.; Qiu X.; Zhu S. Lignin: A Nature-Inspired Sun Blocker for Broad-Spectrum Sunscreens. Green Chem. 2015, 17 (1), 320–324. 10.1039/C4GC01333F.

[ref15] Thalakkale Veettil U.; Moreno A.; Huertas-Alonso A. J.; Morsali M.; Pylypchuk I. V.; Liu L.-Y.; Sipponen M. H. Mechanically Recyclable Melt-Spun Fibers from Lignin Esters and Iron Oxide Nanoparticles: Towards Circular Lignin Materials. Green Chem. 2023, 25, 10424 10.1039/D3GC02381H. 38089756 PMC10711735

[ref16] Pylypchuk I.; Sipponen M. H. Organic Solvent-Free Production of Colloidally Stable Spherical Lignin Nanoparticles at High Mass Concentrations. Green Chem. 2022, 24 (22), 8705–8715. 10.1039/D2GC02316D.

[ref17] Zhang Y.; Naebe M. Lignin: A Review on Structure, Properties, and Applications as a Light-Colored UV Absorber. ACS Sustainable Chem. Eng. 2021, 9 (4), 1427–1442. 10.1021/acssuschemeng.0c06998.

[ref18] Chen M.; Li Y.; Liu H.; Zhang D.; Shi Q.-S.; Zhong X.-Q.; Guo Y.; Xie X.-B. High Value Valorization of Lignin as Environmental Benign Antimicrobial. Materials Today Bio 2023, 18, 100520 10.1016/j.mtbio.2022.100520. PMC9800644 36590981

[ref19] Donnelly P. K.; Entry J. A.; Crawford D. L.; Cromack K. Cellulose and Lignin Degradation in Forest Soils: Response to Moisture, Temperature, and Acidity. Microb Ecol 1990, 20 (1), 289–295. 10.1007/BF02543884. 24193981

[ref20] Yu X.; Chen S.; Wang W.; Deng T.; Wang H. Empowering Alkali Lignin with High Performance in Pickering Emulsion by Selective Phenolation for the Protection and Controlled-Release of Agrochemical. Journal of Cleaner Production 2022, 339, 130769 10.1016/j.jclepro.2022.130769.

[ref21] Beckers S.; Peil S.; Wurm F. R. Pesticide-Loaded Nanocarriers from Lignin Sulfonates—A Promising Tool for Sustainable Plant Protection. ACS Sustainable Chem. Eng. 2020, 8 (50), 18468–18475. 10.1021/acssuschemeng.0c05897. 33381356 PMC7756456

[ref22] Liang W.; Zhang J.; Wurm F. R.; Wang R.; Cheng J.; Xie Z.; Li X.; Zhao J. Lignin-Based Non-Crosslinked Nanocarriers: A Promising Delivery System of Pesticide for Development of Sustainable Agriculture. Int. J. Biol. Macromol. 2022, 220, 472–481. 10.1016/j.ijbiomac.2022.08.103. 35987356

[ref23] Andeme Ela R. C.; Tajiri M.; Newberry N. K.; Heiden P. A.; Ong R. G. Double-Shell Lignin Nanocapsules Are a Stable Vehicle for Fungicide Encapsulation and Release. ACS Sustainable Chem. Eng. 2020, 8 (46), 17299–17306. 10.1021/acssuschemeng.0c06686.

[ref24] Liu J.; Wang X.; Chang J.; Du P.; Wu J.; Hou R.; Zhu S.; Liu P.; Miao X.; Zhang P.; Zhang Z. Green Synthesized Lignin Nanoparticles for the Sustainable Delivery of Pyraclostrobin to Control Strawberry Diseases Caused by Botrytis Cinerea. Int. J. Biol. Macromol. 2024, 274, 133488 10.1016/j.ijbiomac.2024.133488. 38944092

[ref25] Ji L.; Liu L.-Y.; Cho M.; Karaaslan M. A.; Renneckar S. Revisiting the Molar Mass and Conformation of Derivatized Fractionated Softwood Kraft Lignin. Biomacromolecules 2022, 23 (3), 708–719. 10.1021/acs.biomac.1c01101. 34968020

[ref26] Koivu K. A. Y.; Sadeghifar H.; Nousiainen P. A.; Argyropoulos D. S.; Sipilä J. Effect of Fatty Acid Esterification on the Thermal Properties of Softwood Kraft Lignin. ACS Sustainable Chem. Eng. 2016, 4 (10), 5238–5247. 10.1021/acssuschemeng.6b01048.

[ref27] Tian R.; Wang C.; Jiang W.; Janaswamy S.; Yang G.; Ji X.; Lyu G. Biodegradable, Strong, and Hydrophobic Regenerated Cellulose Films Enriched with Esterified Lignin Nanoparticles. Small 2024, 20 (33), 2309651 10.1002/smll.202309651. 38530065

[ref28] Pylypchuk I. V.; Karlsson M.; Lindén P. A.; Lindström M. E.; Elder T.; Sevastyanova O.; Lawoko M. Molecular Understanding of the Morphology and Properties of Lignin Nanoparticles: Unravelling the Potential for Tailored Applications. Green Chem. 2023, 25 (11), 4415–4428. 10.1039/D3GC00703K. 37288453 PMC10243429

[ref29] Porto D. D. S.; Estevão B. M.; Pincela Lins P. M.; Rissi N. C.; Zucolotto V.; Da Silva M. F. G. F. Orange Trunk Waste-Based Lignin Nanoparticles Encapsulating Curcumin as a Photodynamic Therapy Agent against Liver Cancer. ACS Appl. Polym. Mater. 2021, 3 (10), 5061–5072. 10.1021/acsapm.1c00822.

[ref30] Iravani S.; Varma R. S. Greener Synthesis of Lignin Nanoparticles and Their Applications. Green Chem. 2020, 22 (3), 612–636. 10.1039/C9GC02835H.

[ref31] Alqahtani M. S.; Alqahtani A.; Kazi M.; Ahmad M. Z.; Alahmari A.; Alsenaidy M. A.; Syed R. Wound-Healing Potential of Curcumin Loaded Lignin Nanoparticles. Journal of Drug Delivery Science and Technology 2020, 60, 102020 10.1016/j.jddst.2020.102020.

[ref32] Yu B.; Cheng J.; Fang Y.; Xie Z.; Xiong Q.; Zhang H.; Shang W.; Wurm F. R.; Liang W.; Wei F.; Zhao J. Multi-Stimuli-Responsive, Topology-Regulated, and Lignin-Based Nano/Microcapsules from Pickering Emulsion Templates for Bidirectional Delivery of Pesticides. ACS Nano 2024, 18, 10031 10.1021/acsnano.3c11621. 38547360

[ref33] Bueno V.; Gao X.; Abdul Rahim A.; Wang P.; Bayen S.; Ghoshal S. Uptake and Translocation of a Silica Nanocarrier and an Encapsulated Organic Pesticide Following Foliar Application in Tomato Plants. Environ. Sci. Technol. 2022, 56 (10), 6722–6732. 10.1021/acs.est.1c08185. 35467849

[ref34] Zhao P.; Cao L.; Ma D.; Zhou Z.; Huang Q.; Pan C. Translocation, Distribution and Degradation of Prochloraz-Loaded Mesoporous Silica Nanoparticles in Cucumber Plants. Nanoscale 2018, 10 (4), 1798–1806. 10.1039/C7NR08107C. 29308814

[ref35] Bianchi G.; Avato P.; Salamini F. Surface Waxes from Grain, Leaves, and Husks of Maize (Zea Mays L.). Cereal Chem. 1984, 61 (1), 45–47.

[ref36] Gad H. A.; Roberts A.; Hamzi S. H.; Gad H. A.; Touiss I.; Altyar A. E.; Kensara O. A.; Ashour M. L. Jojoba Oil: An Updated Comprehensive Review on Chemistry, Pharmaceutical Uses, and Toxicity. Polymers 2021, 13 (11), 1711 10.3390/polym13111711. 34073772 PMC8197201

[ref37] Busson-Breysse J.; Farines M.; Soulier J. Jojoba Wax: Its Esters and Some of Its Minor Components. J. Americ Oil Chem. Soc. 1994, 71 (9), 999–1002. 10.1007/BF02542268.

[ref38] Gee P. T. Analytical Characteristics of Crude and Refined Palm Oil and Fractions. Euro J. Lipid Sci. Tech 2007, 109 (4), 373–379. 10.1002/ejlt.200600264.

[ref39] Barrera-Arellano D.; Badan-Ribeiro A. P.; Serna-Saldivar S. O. Corn Oil: Composition, Processing, and Utilization. Corn 2019, 593–613. 10.1016/B978-0-12-811971-6.00021-8.

[ref40] Machado T. O.; Grabow J.; Sayer C.; De Araújo P. H. H.; Ehrenhard M. L.; Wurm F. R. Biopolymer-Based Nanocarriers for Sustained Release of Agrochemicals: A Review on Materials and Social Science Perspectives for a Sustainable Future of Agri- and Horticulture. Adv. Colloid Interface Sci. 2022, 303, 102645 10.1016/j.cis.2022.102645. 35358807

[ref41] Moreno A.; Liu J.; Gueret R.; Hadi S. E.; Bergström L.; Slabon A.; Sipponen M. H. Unravelling the Hydration Barrier of Lignin Oleate Nanoparticles for Acid- and Base-Catalyzed Functionalization in Dispersion State. Angew. Chem. Int. Ed 2021, 60 (38), 20897–20905. 10.1002/anie.202106743. PMC8518943 34196470

[ref42] Rostamitabar M.; Abdelgawad A. M.; Jockenhoevel S.; Ghazanfari S. Drug-Eluting Medical Textiles: From Fiber Production and Textile Fabrication to Drug Loading and Delivery. Macromol. Biosci. 2021, 21 (7), 2100021 10.1002/mabi.202100021. 33951278

[ref43] Malekjani N.; Jafari S. M. Modeling the Release of Food Bioactive Ingredients from Carriers/Nanocarriers by the Empirical, Semiempirical, and Mechanistic Models. Comp Rev. Food Sci. Food Safe 2021, 20 (1), 3–47. 10.1111/1541-4337.12660. 33443795

[ref44] Kunz M.; Staiger S.; Burghardt M.; Popp C.; George N.; Roberts K.; Riederer M. Diffusion Kinetics of Active Ingredients and Adjuvants in Wax Films: An Attenuated Total Reflection-Infrared Spectroscopy Study of a Leaf Surface Model. ACS Agric. Sci. Technol. 2022, 2 (3), 625–638. 10.1021/acsagscitech.2c00054.

[ref45] Avellan A.; Yun J.; Zhang Y.; Spielman-Sun E.; Unrine J. M.; Thieme J.; Li J.; Lombi E.; Bland G.; Lowry G. V. Nanoparticle Size and Coating Chemistry Control Foliar Uptake Pathways, Translocation, and Leaf-to-Rhizosphere Transport in Wheat. ACS Nano 2019, 13 (5), 5291–5305. 10.1021/acsnano.8b09781. 31074967

[ref46] Corredor E.; Testillano P. S.; Coronado M.-J.; González-Melendi P.; Fernández-Pacheco R.; Marquina C.; Ibarra M. R.; De La Fuente J. M.; Rubiales D.; Pérez-de-Luque A.; Risueño M.-C. Nanoparticle Penetration and Transport in Living Pumpkin Plants: In Situsubcellular Identification. BMC Plant Biol. 2009, 9 (1), 45 10.1186/1471-2229-9-45. 19389253 PMC2680855

[ref47] Hu P.; An J.; Faulkner M. M.; Wu H.; Li Z.; Tian X.; Giraldo J. P. Nanoparticle Charge and Size Control Foliar Delivery Efficiency to Plant Cells and Organelles. ACS Nano 2020, 14 (7), 7970–7986. 10.1021/acsnano.9b09178. 32628442

[ref48] Beaumont M.; Tardy B. L.; Reyes G.; Koso T. V.; Schaubmayr E.; Jusner P.; King A. W. T.; Dagastine R. R.; Potthast A.; Rojas O. J.; Rosenau T. Assembling Native Elementary Cellulose Nanofibrils via a Reversible and Regioselective Surface Functionalization. J. Am. Chem. Soc. 2021, 143 (41), 17040–17046. 10.1021/jacs.1c06502. 34617737 PMC8532154

[ref49] Li M.; Zhu L.; Xiao H.; Shen T.; Tan Z.; Zhuang W.; Xi Y.; Ji X.; Zhu C.; Ying H. Design of a Lignin-Based Versatile Bioreinforcement for High-Performance Natural Rubber Composites. ACS Sustainable Chem. Eng. 2022, 10 (24), 8031–8042. 10.1021/acssuschemeng.2c02113.

[ref50] Dash S.; Murthy P. N.; Nath L.; Chowdhury P. Kinetic Modeling on Drug Release from Controlled Drug Delivery Systems. Acta Pol. Pharm. - Drug Res. 2010, 67 (3), 217–223. 20524422

[ref51] Mathematical Models of Drug Release. In Strategies to Modify the Drug Release from Pharmaceutical Systems; Elsevier, 2015; pp 63–86.

[ref52] Easley A. D.; Ma T.; Eneh C. I.; Yun J.; Thakur R. M.; Lutkenhaus J. L. A Practical Guide to Quartz Crystal Microbalance with Dissipation Monitoring of Thin Polymer Films. J. Polym. Sci. 2022, 60 (7), 1090–1107. 10.1002/pol.20210324.

[ref53] Zhao X.; Zeng Q.; Yang S.; He F.; Qin W.; Wang Z.; Mai K.; Yu G.; Huang J.; Li J.; Feng Y. Light-Tuning the Host–Guest Interfacial Recognition of Alginate-Based Amphiphiles for Oil-in-Water Droplet Deposition. Chemical Engineering Journal 2023, 463, 142362 10.1016/j.cej.2023.142362.

[ref54] Fagerström A.; Kocherbitov V.; Westbye P.; Bergström K.; Arnebrant T.; Engblom J. Surfactant Softening of Plant Leaf Cuticle Model Wax – A Differential Scanning Calorimetry (DSC) and Quartz Crystal Microbalance with Dissipation (QCM-D) Study. J. Colloid Interface Sci. 2014, 426, 22–30. 10.1016/j.jcis.2014.03.064. 24863760

[ref55] Argyropoulos D. S.; Pajer N.; Crestini C. Quantitative ^31^P NMR Analysis of Lignins and Tannins. JoVE 2021, 174, 62696 10.3791/62696. 34398158

[ref56] Granata A.; Argyropoulos D. S. 2-Chloro-4,4,5,5-Tetramethyl-1,3,2-Dioxaphospholane, a Reagent for the Accurate Determination of the Uncondensed and Condensed Phenolic Moieties in Lignins. J. Agric. Food Chem. 1995, 43 (6), 1538–1544. 10.1021/jf00054a023.

[ref57] Pu Y.; Cao S.; Ragauskas A. J. Application of Quantitative 31P NMR in Biomass Lignin and Biofuel Precursors Characterization. Energy Environ. Sci. 2011, 4 (9), 3154 10.1039/c1ee01201k.

