# Supplementary material for: Esterified Lignin Nanoparticles for Targeted Chemical Delivery in Plant Protection
Source: ACS Appl Mater Interfaces. 2024 Dec 21;17(1):1931–41. doi: 10.1021/acsami.4c16912 (PMC11783360; doi:10.1021/acsami.4c16912)
Supplement: Supplementary file 1 — am4c16912_si_001.pdf [file am4c16912_si_001.pdf]

SUPPORTING INFORMATION

Esterified lignin nanoparticles for targeted chemical delivery in plant protection

*Matilda Andersson [a], Ievgen V. Pylypchuk [a], Alexandros Efraim Alexakis [a], Li-Yang Liu [a, ‡], Mika H. Sipponen [a, *]*

[a] Department of Materials and Environmental Chemistry, Stockholm University, Svante Arrhenius väg 16C, 106 91 Stockholm, Sweden

[‡] Present address: Department of Chemistry and Chemical Engineering, Chalmers University of Technology, Kemigården 4, 412 58 Gothenburg, Sweden

**Corresponding author: mika.sipponen@mmk.su.se*

1. Experimental Section

1.1 Extraction of wax from *Zea maize L.* leaves

The collected leaves were cleaned with water and gentle scrubbing and then allowed to fully dry at room temperature. Thereafter, the leaves were cut into bigger pieces and submerged in cyclohexane for either 1, 2, 4, 8, or 24 hours. Afterward, solutions were filtered, and the solvent was evaporated and collected using a rotary evaporator.

1.1.1 Characterisation of extracted material

Attenuated Total Reflectance Fourier Transform Infrared (ATR-FTIR) Spectroscopy was used for the characterisation of functional groups of the wax extracted at different times and to control changes in composition over time. Varian 610-IR Spectrometer with a diamond ATR Optics were used and measurements were taken from 400 to 4000 cm^{-1} with a total of 32 scans.

Differential Scanning Calorimetry (DSC) was used for the determination of the melting point of the extracted wax. This was done using Netzsch DSC 214 Polyma instrument. The heating and cooling rate used for the program was 10 ºC min^{-1}, and N_2 was used as the purge gas. The program started at 10 ºC and increased to 100 ºC, followed by cooling to 20 ºC.

1.1.2 Quantification of chlorophyll content

To quantify the chlorophyll extracted with the wax, each batch of dried wax was dissolved in acetone and analysed using Genesys 150 UV-vis spectrophotometer (Thermoscientific, USA) at λ = 662 nm (chlorophyll a) and λ = 645 nm (chlorophyll b). Thereafter, the total mass of chlorophyll in the extracted sample was calculated using formula S1.

$$m\ (mg) = \left(\frac{A_{662\ \mathrm{nm}}}{\varepsilon_{662\ \mathrm{nm}} \times l}\right) \times V + \left(\frac{A_{645\ \mathrm{nm}}}{\varepsilon_{645\ \mathrm{nm}} \times l}\right) \times V \qquad \text{(S1)}$$

A is the absorbance obtained during measurements of chlorophyll a and b, ε is the specific adsorption coefficient for chlorophyll a (88.88 L g^{-1} cm^{-1}) and b (56.11 L g^{-1} cm^{-1}) in acetone,[1] l is the path length (1 cm) and V is the volume of acetone.

2. Results

Table S1. Summary of ^{31}P NMR results of SKL and EL. The total hydroxyl groups represent the sum of the aliphatic and aromatic hydroxyl groups.

	**Aliphatic OH (mmol g^{-1})**	**Aromatic OH (mmol g^{-1})**	**Total OH (mmol g^{-1})**	**COOH (mmol g^{-1})**	**DE (%)**
**SKL**	2.0 ± 0.26	4.5 ± 0.41	6.47 ± 0.66	0.55 ± 0.11	-
**EL$_{40\ °C,\ 3\ h}$**	0.65 ± 0.13	2.0 ± 0.11	2.70 ± 0.22	3.06 ± 0.40	58
**EL$_{60\ °C,\ 3\ h}$**	0.71 ± 0.12	2.0 ± 0.16	2.74 ± 0.21	2.95 ± 0.30	57
**EL$_{40\ °C,\ 24\ h}$**	0.68 ± 0.053	2.0 ± 0.10	2.63 ± 0.049	3.59 ± 0.33	58

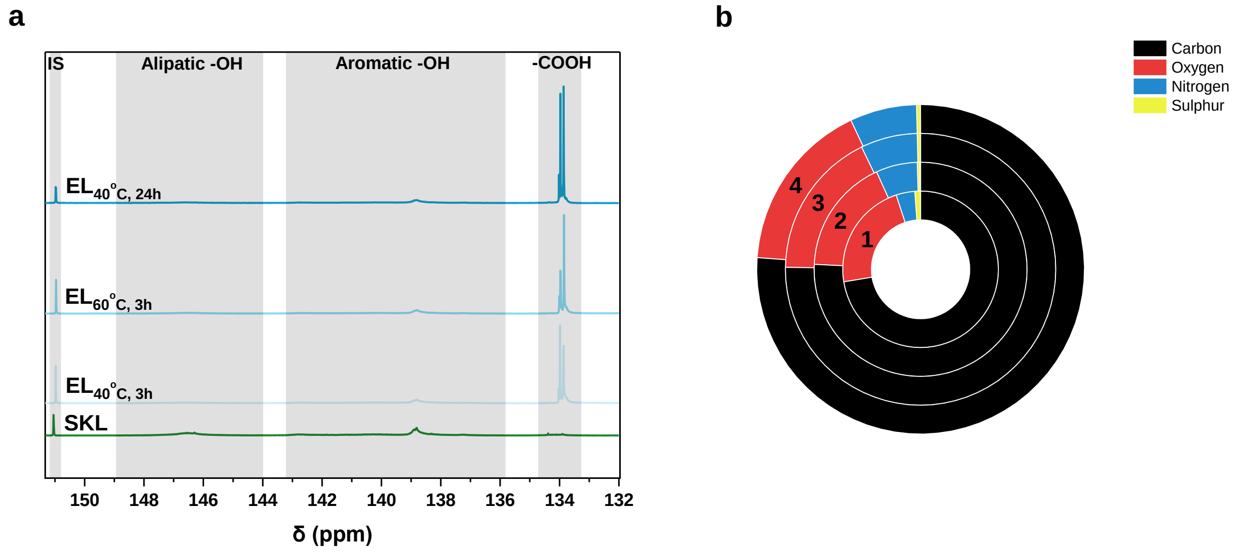

Figure S1. Characterisation of esterified product. (a) ^{31}P NMR spectra of SKL and the three different batches of EL, and (b) SEM-EDS results for SKL and the three different batches of EL presented in atomic percentage. 1 represents the SKL, 2 is EL synthesised at 40 ºC for 3 hours, 3 is EL synthesised at 60 ºC for 3 hours, and 4 is EL synthesised at 40 ºC for 24 hours.

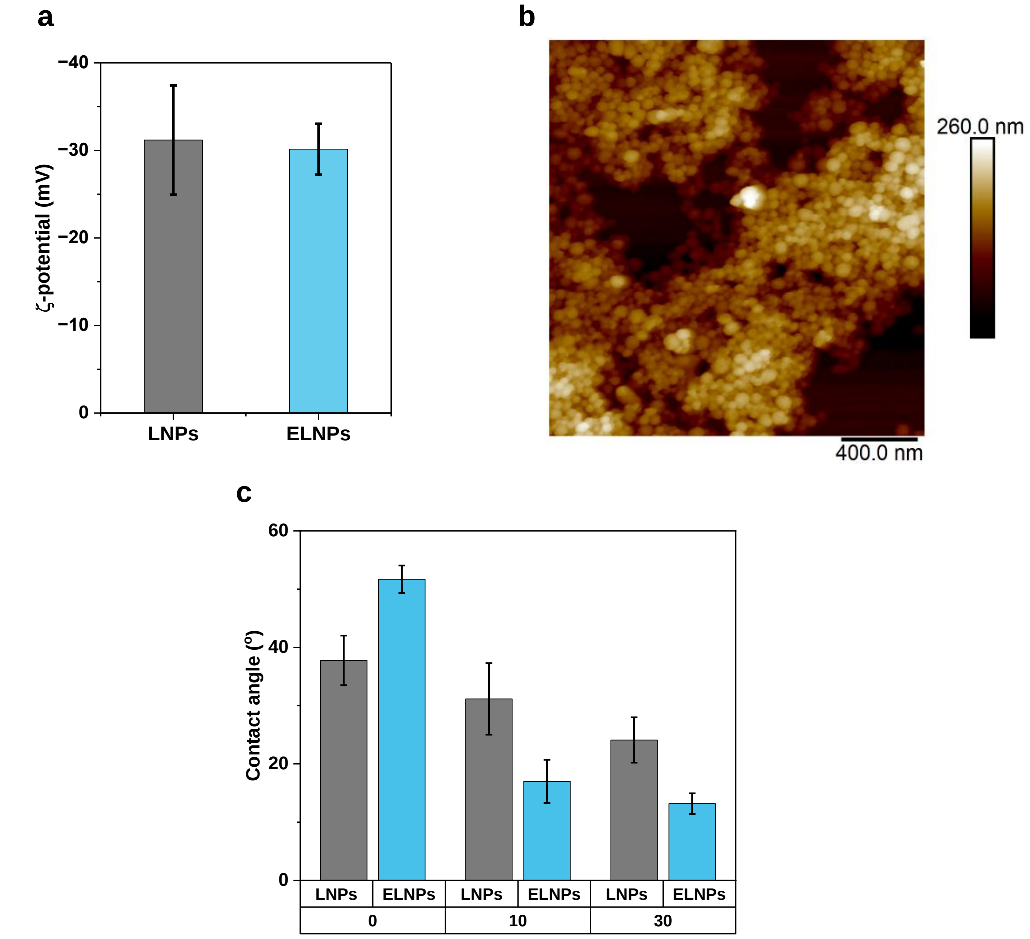

Figure S2. Characterisation of nanoparticles. (a) ζ-potential for LNPs and ELNPs, (b) AFM-height image of the LNPs, (c) water contact angle measurement of LNP and ELNP coatings.

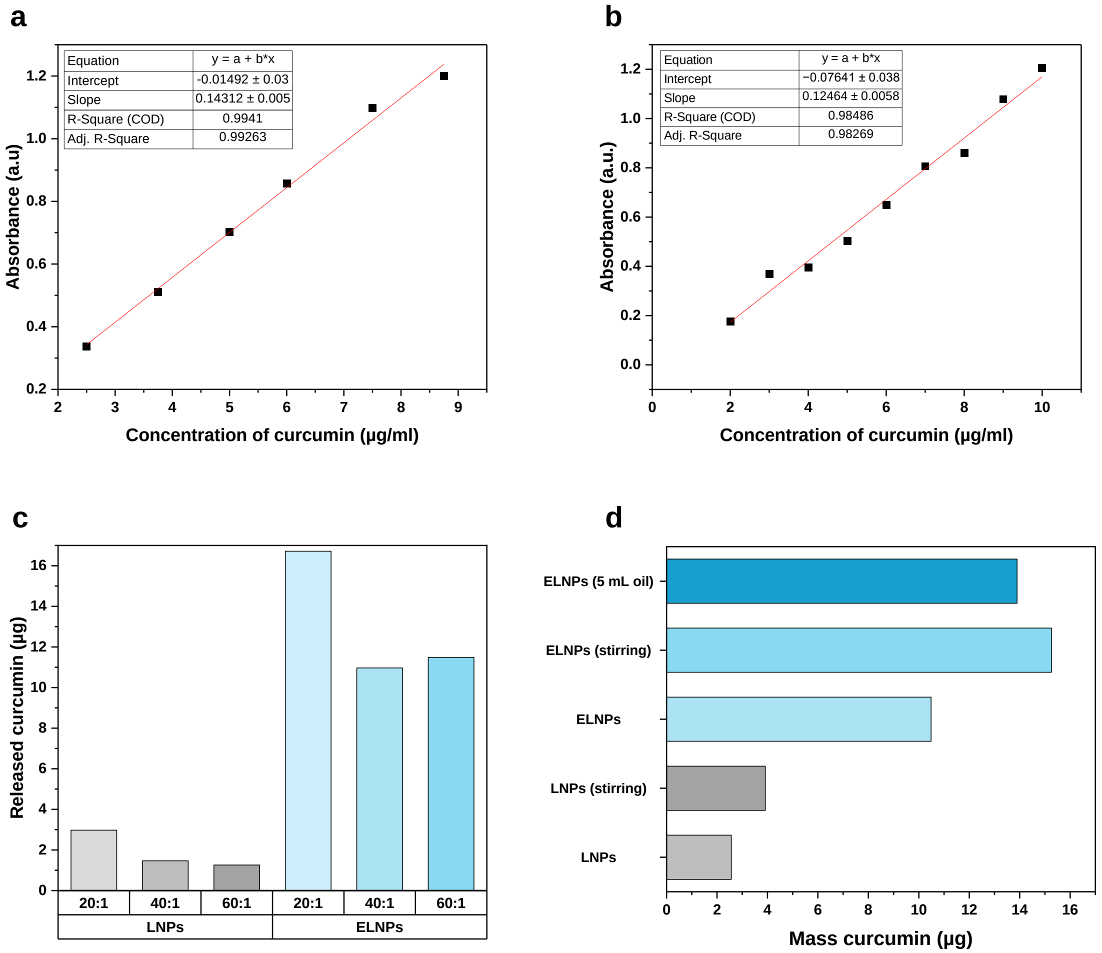

Figure S3. Release of curcumin. (a) Calibration curve for curcumin in 3:1 acetone/water (mass ratio), (b) calibration curve of curcumin in jojoba oil, (c) maximum release obtained of the curcumin in jojoba oil after 1 hour of sonication, and (d) mass released after 30 days as a result of stirring or increased oil volume compared to the sample used for test of release kinetics.

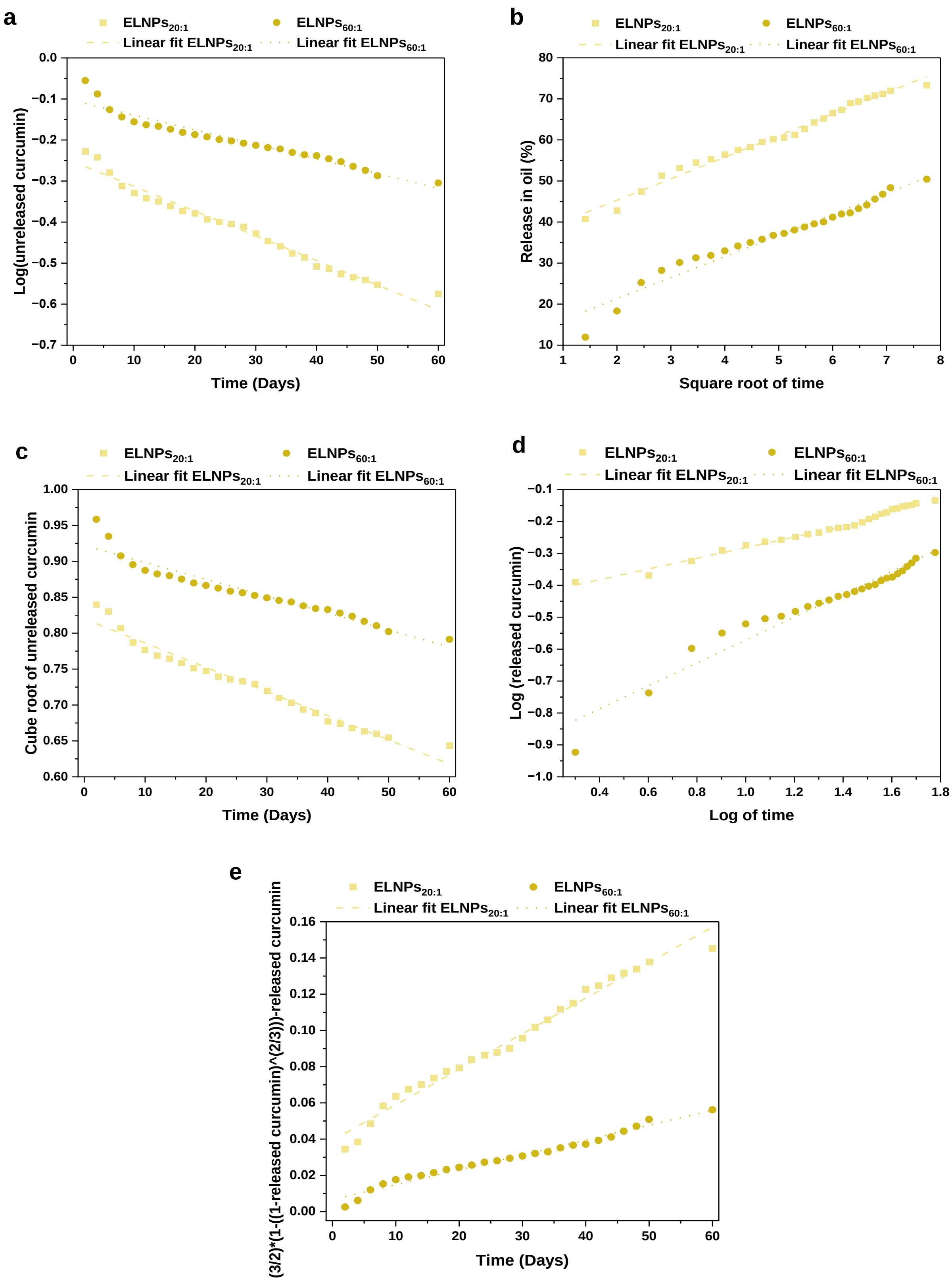

Figure S4. Model testing of release kinetics. (a) First-order model, (b) Higuch model, (c) Hixon-

Corwell model, (d) Korsmeyer-Peppas model, and (e) Baker-Lonsdale model.

Table S2. Summary of model testing for the release kinetics. The correlation coefficient (R^2) and the n value for the Korsmeyer-Peppas model are presented.

	Release kinetics model					
**Lignin:curcumin mass ratio**	**First order**	**Hiuguchi**	**Hixon-Corwell**	**Korsmeyer-Peppas**		**Baker-Lonsdale**
	**R^2**	**R^2**	**R^2**	**R^2**	**n**	**R^2**
**20:1**	0.9744	0.9862	0.9644	0.9802	0.17	0.9812
**60:1**	0.9299	0.9552	0.9162	0.9480	0.36	0.9754

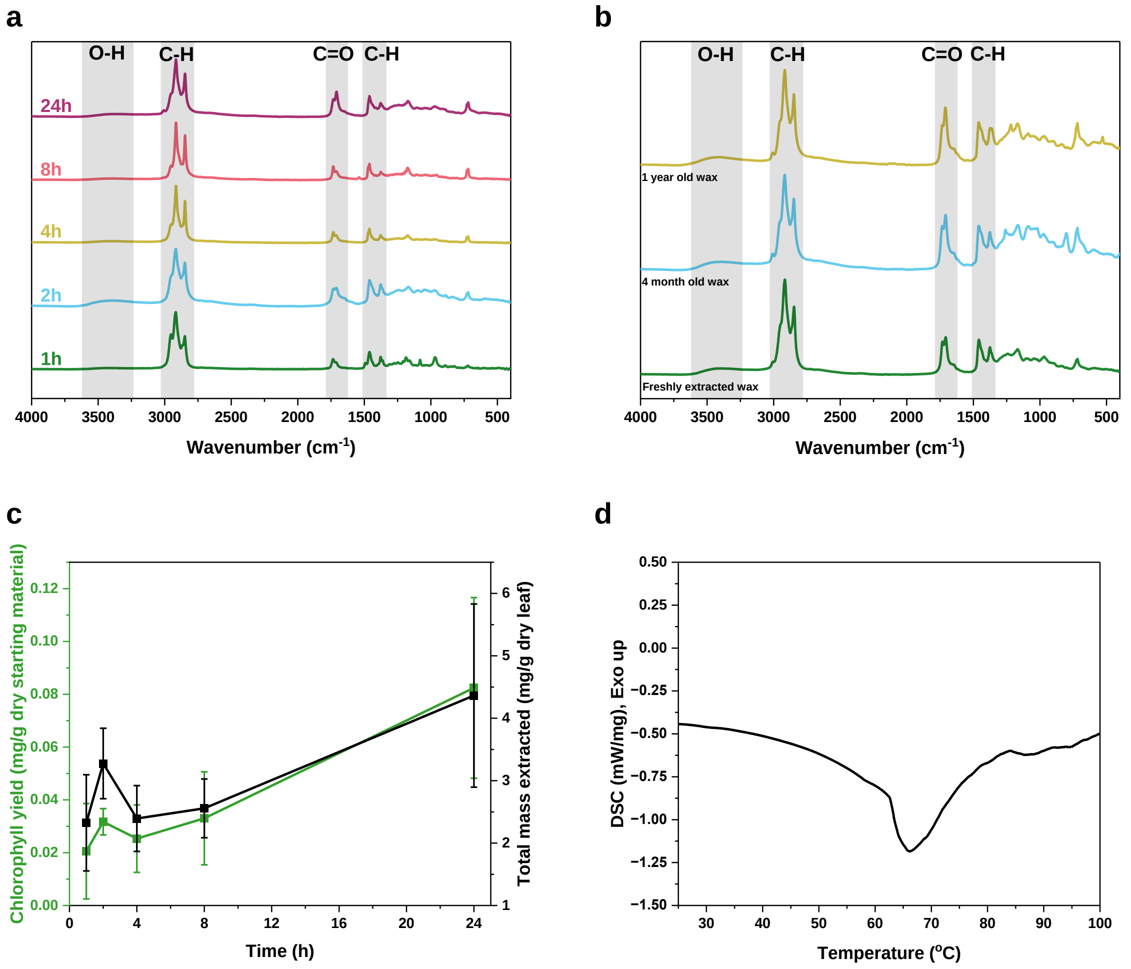

Figure S5. Characterisation of extracted wax. (a) ATR-FTIR of the wax extracted for 1, 2, 4, 8 or 24 hours. (b) Control of stability of extracted wax, (c) quantification of chlorophyll content in extracted mass and mass extracted for leaves. (d) DSC thermogram of extracted wax.

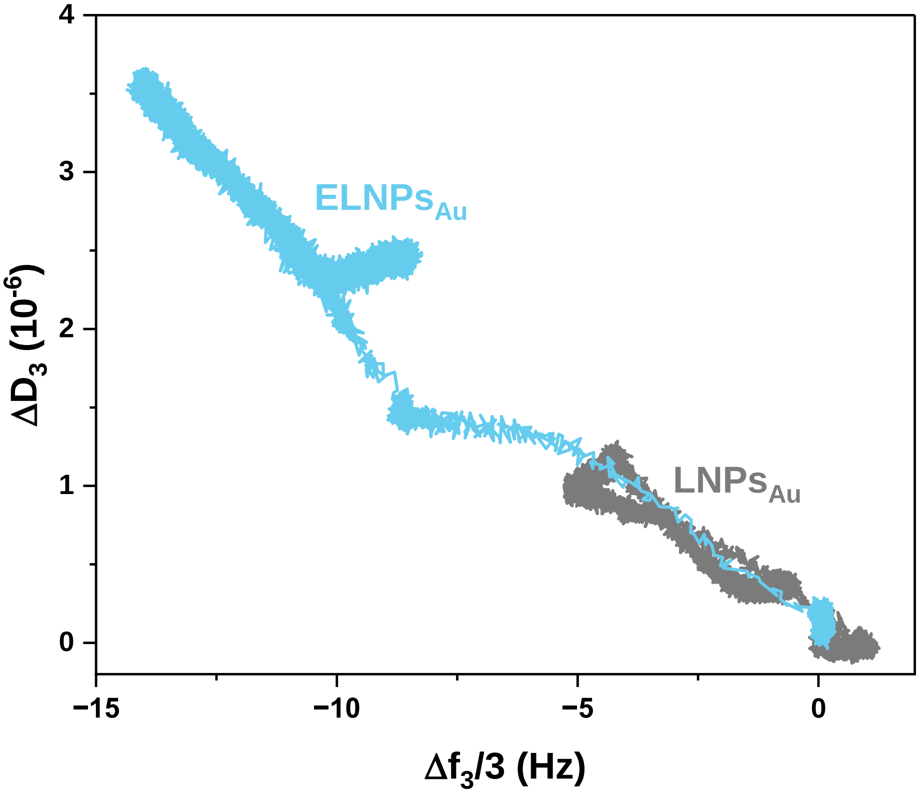

Figure S6. Dissipation versus frequency plot for the interaction of LNPs/ELNPs on clean gold sensor obtained by QCM-D.

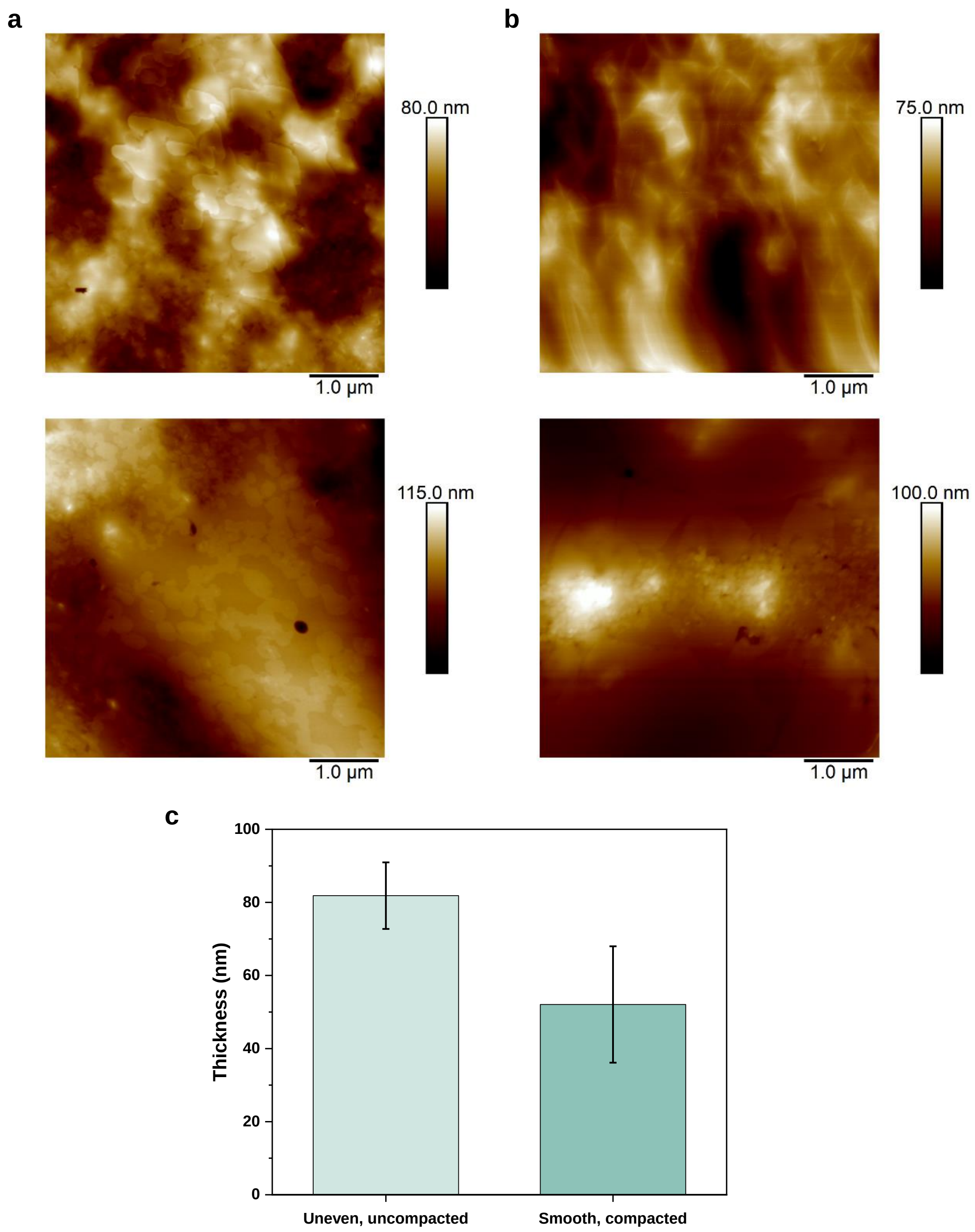

Figure S7. AFM-height images of wax coated sensor with (a) uneven, uncomapcted and (b) smooth, compacted wax film. (c) Estimation of coating of the thickness with the error bar representing +/- one standard deviation (n = 10 for uneven, uncompacted wax film and n = 15 for smooth, compacted wax film).

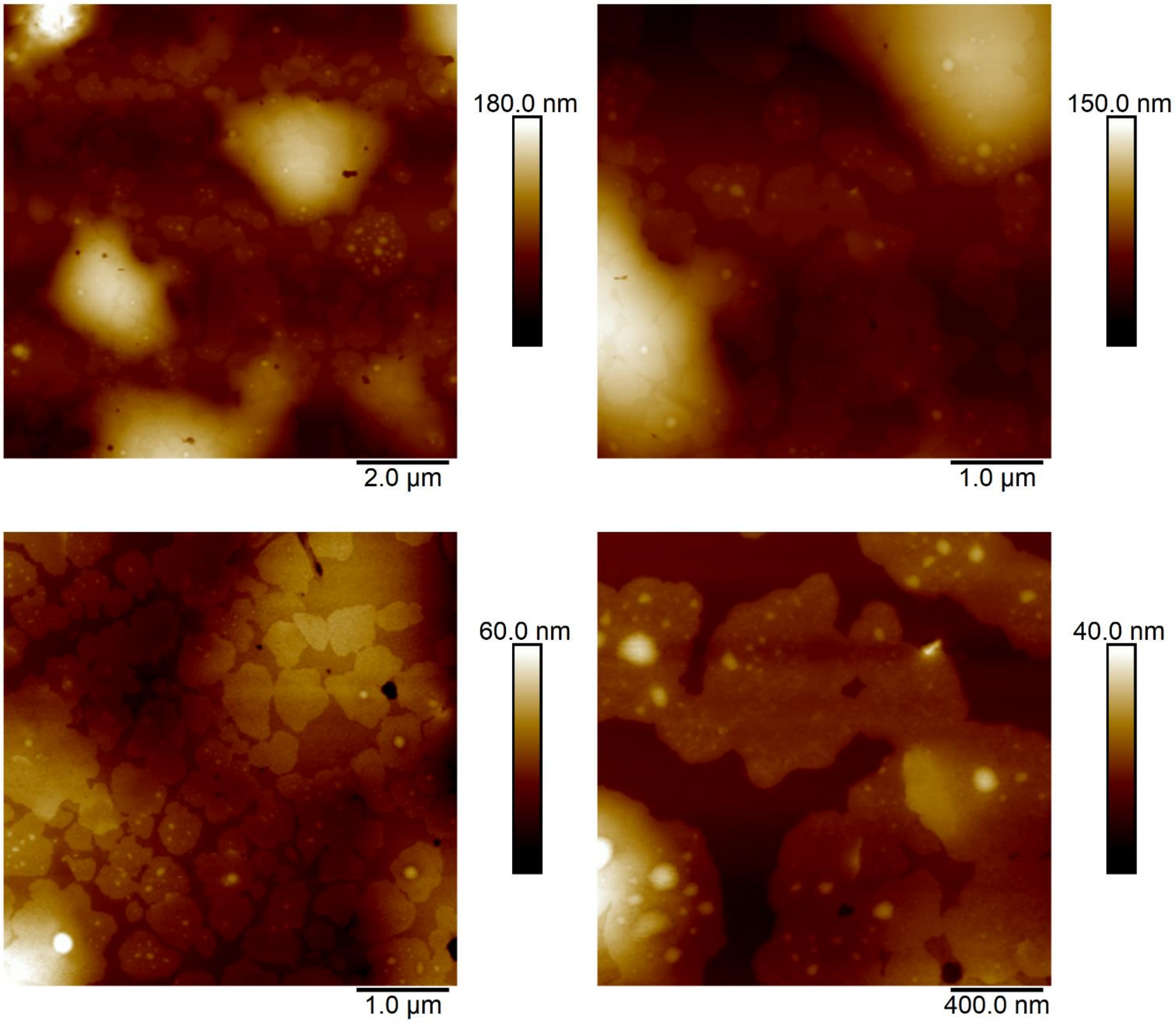

Figure S8. AFM-height images of wax-coated silicon wafer.

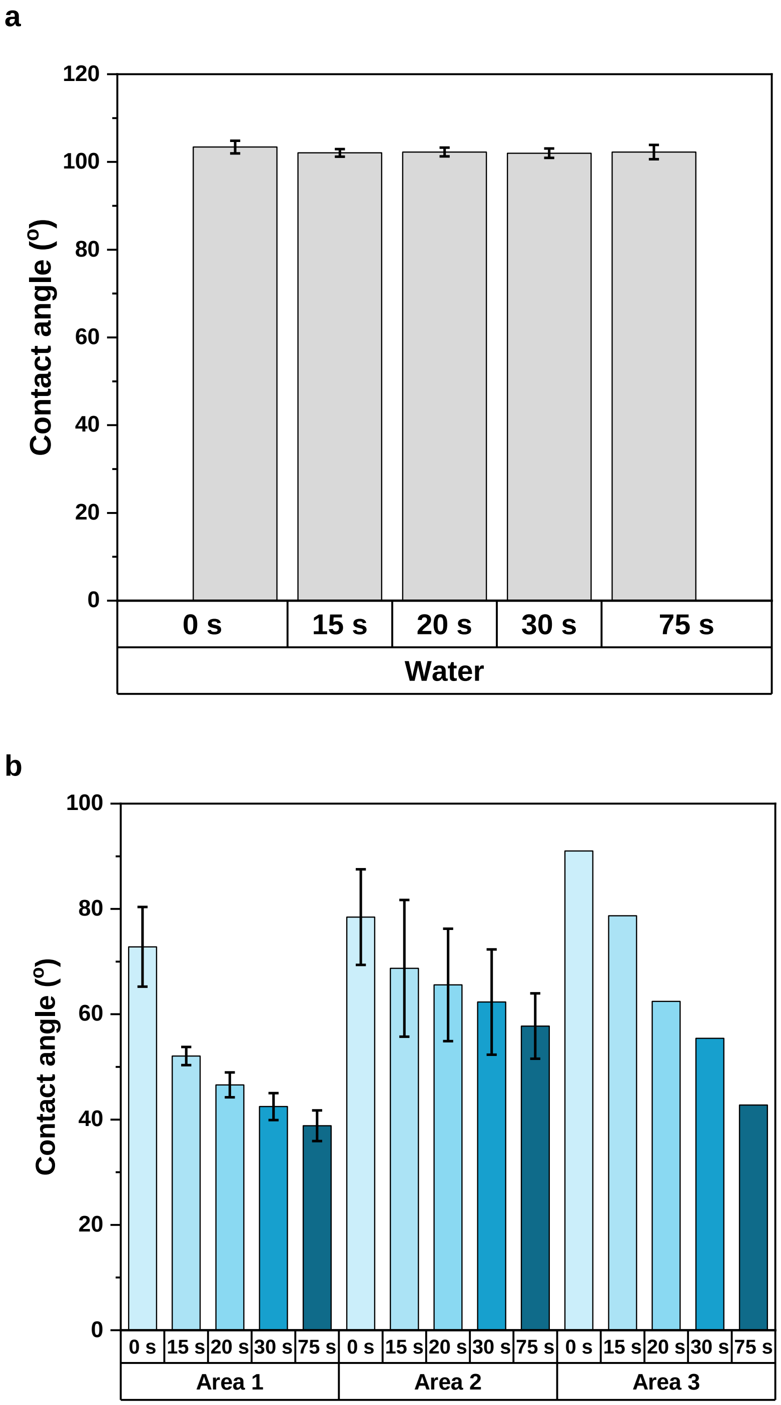

Figure S9. Contact angle of corn leaf with (a) water and (b) ELNPs on different areas of the leaf. Error bars in (a) and (b) is based on +/- one standard deviation (n = 5 for (a) and n = 5, n = 3 and n = 1 for the respective area going left to right in (b)).

References

(1) Lichtenthaler, H. K.; Wellburn, A. R. Determinations of Total Carotenoids and Chlorophylls a and b of Leaf Extracts in Different Solvents. *Biochemical Society Transactions* **1983**, *11* (5), 591–592. https://doi.org/10.1042/bst0110591.
